# Singing interventions for people living with Parkinson’s: a systematic review and meta-analysis

**DOI:** 10.1136/bmjopen-2024-089154

**Published:** 2025-11-24

**Authors:** J Yoon Irons, David Sheffield

**Affiliations:** 1 School of Psychology, University of Derby, Derby, UK

**Keywords:** Neurology, Parkinson-s disease, Quality of Life

## Abstract

**Abstract:**

**Objectives:**

To evaluate the effects of singing interventions on well-being, mental health and communication, motor and respiratory functions through meta-analysis and to examine the practices used in the singing interventions.

**Design:**

Systematic review and meta-analysis.

**Inclusion criteria:**

Both randomised and non-randomised studies, involving participants living with Parkinson’s and receiving singing interventions.

**Data sources:**

Four databases (CINAHL, Medline, PsycINFO, Web of Science) and Google Scholar were searched. The last search was conducted on 3 April 2025.

**Primary and secondary outcome measures:**

Eligible studies reported on the following outcomes: quality of life, voice-related acoustic measures, respiratory function, mental health and motor function. The risk of bias was assessed using the Downs and Black Quality checklist for controlled studies and national heart, lung, blood institute study quality assessment tool for non-controlled studies.

**Analysis:**

Meta-analyses were conducted to pool effect sizes across included studies using random-effects models. All analyses were performed using Meta-Essentials. Additionally, key elements of singing practices were narratively synthesised.

**Results:**

23 studies (3 randomised controlled trials (RCTs), 20 non-RCTs) involving 540 participants were included in the analysis. Common elements of singing intervention practices included breathing exercises, vocal warm-ups and singing participants’ preferred songs, which are largely led by music therapists.Three meta-analyses based on RCTs were conducted on clinical outcome measures; results suggest that singing was favoured in only one outcome measure, vocal loudness during sustained vowel production (standard mean difference (SMD)=0.67, 95% CI 0.29 to 1.05, I^2^=0%). However, the certainty of this evidence is very low due to a high risk of bias, imprecision and indirectness. Further, when combining results from RCTs and non-RCTs, positive changes for three further outcome measures were observed: maximum phonation time (k=11, n=157, SMD=0.38, 95% CI 0.18 to 0.59, I^2^=23.48%), vocal loudness of sustained vowel (k=8, n=99, SMD=0.50, 95% CI 0.14 to 0.86, I^2^=50.96%) and a respiratory function measure of maximal inspiration pressure (k=4, n=65, SMD=0.46, 95% CI 0.07 to 0.85, I^2^=0%). However, these findings are largely based on non-controlled studies with very low quality of evidence.

**Conclusions:**

Singing interventions may support people living with Parkinson’s, but due to insufficient high-quality evidence, we are unable to determine the effects of singing interventions. We discuss implications for future work and practice, emphasising that more robust RCTs are needed.

**STRENGTHS AND LIMITATIONS OF THIS STUDY**

- Up-to-date systematic review and meta-analysis.
- Including three randomised controlled studies.
- Limited meta-analysis performed due to limited evidence.
- Synthesis of key elements of singing interventions.
- Only studies in English included.

## Introduction

 Parkinson’s disease or Parkinson’s is a complex chronic neurological condition that impacts on a range of body functions. Parkinson’s is characterised primarily by progressively worsening symptoms of movement abnormalities (e.g., bradykinesia, rigidity and resting tremor). People living with Parkinson’s (PLwP) can also experience sleep disturbance, constipation, hyposmia, depression and anxiety.^1^ Furthermore, Parkinson’s affects speech function due to disturbances in the muscular control of the speech mechanism.^2^ The onset of Parkinson’s is often unrecognised, and it may take years for PLwP to receive a diagnosis.^3^

Currently, there is no known cure for Parkinson’s and treatments for Parkinson’s are complex. They include medications, surgical procedures and rehabilitation therapies such as physical, occupational and speech and language therapy.^4^ Rehabilitation therapies focus on the restoration of mobility and balance (physiotherapy), improvement of personal self‐care activities (occupational therapy) and alleviation of communication difficulties (speech and language therapy).^4^ Current pharmacotherapy can reduce Parkinson’s symptoms but is associated with unwanted side effects, such as dyskinesia, dystonia, motor fluctuations, oedema, somnolence, dizziness and hallucinations.^5^ These complications, and the increasing level of disability, significantly influence quality of life (QoL) in PLwP.^6^ Active group-based performing arts interventions, such as dancing and singing, have gained popularity among PLwP. They provide enjoyable group activities, and some studies suggest potential improvements in clinical outcomes; for example, dance interventions have shown benefits for motor function and singing interventions have shown benefits for speech function.^7^ Further, some studies suggest singing interventions may enhance QoL,^8 9^ emotional and social wellbeing^10^ as well as maintain and/or improve speech function in PLwP.^11^ Singing interventions, commonly delivered in a group setting, can be motivating, enjoyable and may offer opportunities to apply necessary voice/speech-related exercises, as singing and speech use the same mechanism.^12^

A previous systematic review on singing interventions for PLwP found a range of benefits but was limited to a narrative synthesis,^11^ and another recent review^7^ reported benefits for QoL and speech function, but no specific meta-analyses were conducted. It is therefore timely to conduct a systematic review of the efficacy and practices of singing interventions for PLwP. We aimed to evaluate the effectiveness of singing interventions for clinical outcome measures, including QoL, well-being, mental health and speech, motor and respiratory functions through meta-analysis. We also aimed to describe the interventions used in the singing studies.

## Methods

The original protocol for the systematic review was published in 2019^13^ and an updated protocol is available via Open Science Framework.^14^

### Searches

We searched the major electronic databases (CINAHL, Medline, PsycINFO and Web of Science) and the first 10 pages of Google Scholar. We used the following relevant Medical Subject Headings (MeSH): Parkinson’s, Parkinsonian Disorders or PD or Parkinsonism; Singing; Voice or vocal exercise or training; Choir; Music therapy (further details are provided in online supplemental file 1). We also inspected references included in the recent systematic reviews on related topics, such as music therapy,^15 16^ the arts,^17^ speech therapy,^18 19^ group-based interventions for Parkinson’s^7^ and neurological rehabilitation.^20^ The last search date was 3 April 2025.

### Types of studies

We included peer-reviewed, empirical studies of singing interventions involving PLwP; these included randomised controlled trials (RCTs), quasi-RCTs, cohort studies and pretest and post-test studies. Additionally, we only included published works in English.

### Types of participants

We included studies involving individuals with medically diagnosed idiopathic Parkinson’s, receiving concurrent treatments. We would not place any restriction on age, sex, ethnicity, drug therapy, other treatments, disease severity or length of diagnosis.

### Types of interventions

We searched for singing interventions (eg, group singing, choir, individual singing training) facilitated by professionals with a relevant qualification (eg, music therapists, professional singing teachers, speech and language therapists, musicians, nurses, occupational therapists or physiotherapists). Studies were required to include at least 2 weeks of singing intervention, based on prior research^21 22^ identifying this duration as a minimum threshold. We included both in-person and online singing.

### Types of outcome measures

#### Primary outcomes

1. QoL: For example, Voice-Related QoL (V-RQoL) (eg, V-RQoL, Voice Handicap Index (VHI)), Parkinson’s related QoL, such as Parkinson’s Disease Questionnaire (PDQ-39 or PDQ-8) or other generic QoL measures, such as WHO Quality of Life Questionnaire-brief version, Short Form-36.
2. Well-being assessments: For example, Warwick-Edinburgh Mental Well-being Scale.

#### Secondary outcomes

1. Voice and communication acoustic outcome measures: for example, acoustic characteristics or standardised quantitative intelligibility assessments.
2. Respiratory function measures: For example, Forced Vital Capacity, Maximal Inspiratory Pressure (MIP) or Maximal Expiratory Pressure (MEP).
3. Mental health measures: For example, Hospital Anxiety and Depression Scale, or Depression Anxiety Stress Scales (DASS).
4. Motor function measures: For example, Unified Parkinson’s Disease Rating Scales motor score, Part III, fall history, walking quantity and quality.

### Adverse effects

We assessed any adverse effects reported in the studies.

### Data collection and analysis

Selection of studies: Two review authors screened articles using titles and abstracts. We then obtained the full-text articles of potentially eligible studies, and two review authors screened these full-text articles. Any disagreements would have been resolved by consulting an independent researcher.

### Data extraction and management

Two review authors independently extracted data onto a data collection form, including citation details, trial setting, inclusion and exclusion criteria, study population, intervention details, outcome measures and results. We resolved any differences in opinion through discussion or, if necessary, through an independent researcher. We contacted study authors for additional information and data if needed.

### Quality assessments of the included studies

Two review authors independently assessed the methodological quality of the included RCTs and controlled studies using a modified Downs and Black Quality Checklist utilised in previous studies.^23^ The modified Downs and Black Quality Checklist consists of 27 questions in relation to reporting (question #1–#10), external validity (question #11–#13), internal validity (question #14–#20), selection bias (question #21–#26) and statistical power (question #27), where only one question instead of two regarding statistical power was asked in this modified version. This was chosen because it can be used for both RCTs and non-RCTs with control groups. For the pretest and post-test studies without a control group, the review authors adopted the National Heart, Lung and Blood Institute (NHLBI) Quality assessment tool,^24^ which consists of 12 questions relating internal validity allowing examination of sample size, blinding assessor and consistency of outcome reporting. Due to using two different tools, quality assessments’ results were not used as a moderator in the meta-analysis.

### Reporting bias assessment

Reporting bias was assessed by funnel plot asymmetry and examination of selective reporting within each included study.

### Measures of treatment effect

Meta-analyses were conducted to pool effect sizes across included studies using random-effects models. All analyses were performed using Meta-Essentials.^25^ Due to the variation in the samples analysed (ie, different measures, different designs), standard mean differences (SMDs) and random effects models were chosen as the most appropriate analytical approach.^26^ Uncertainty for findings is expressed in the form of 95% CIs. Evaluation of effect sizes followed Cohen’s rules of thumb, with 0.2 considered a small effect, 0.5 a moderate effect and 0.8 a large effect.^27^ Additionally, Grading of Recommendations Assessment, Development and Evaluation assessment (risk of bias, inconsistency, indirectness, imprecision and publication bias) is used to express the certainty of evidence in the Summary of Findings Table. Heterogeneity was investigated using Q and Higgins I^2^ statistics.^28^ Publication bias was examined informally by using funnel plots of effect size against SE for asymmetry and formally by using Begg and Mazumdar’s rank correlations,^29^ and Egger’s regression intercept test. Additionally, possible bias was determined using the trim-and-fill method. Further, to check any selective reporting of outcomes, we compared trial protocols with published papers, where possible.

### Patient and public involvement

Patient and public involvement was not conducted for this systematic review, as the nature of the work did not necessitate engagement with patients or the public.

## Results

### Search results and included studies

**Figure 1:**
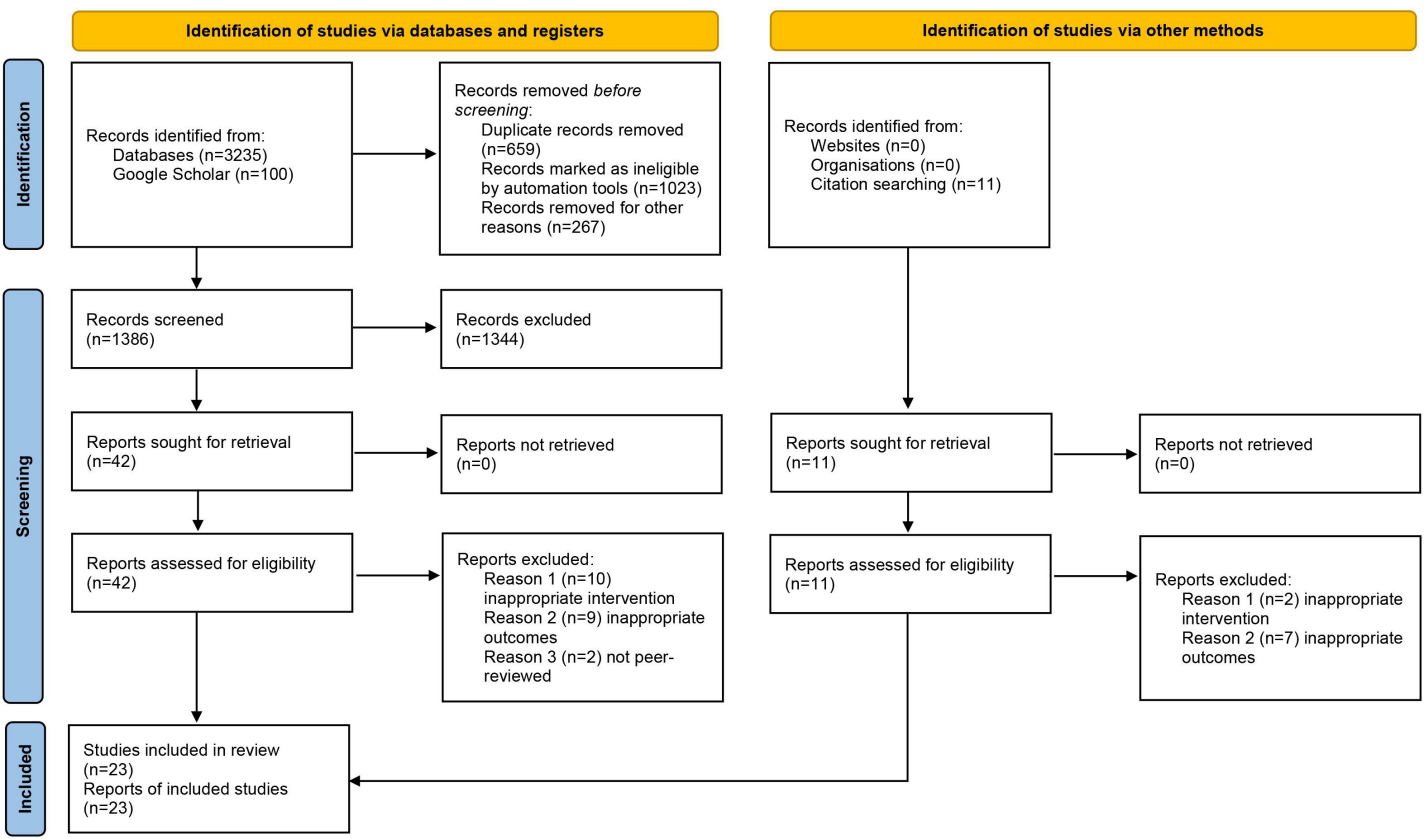
PRISMA flow diagram. PRISMA, Preferred Reporting Items for Systematic Reviews and Meta-Analyses.

Through electronic database and citation searches, a total of 3235 hits were identified. Rayyan, a review management platform, was used to screen studies. After deleting duplicates, irrelevant records and screening abstracts and full texts, 23 studies met inclusion criteria (figure 1). Studies were excluded if (1) they did not include singing interventions of a minimum of 2 weeks, (2) they did not have appropriate outcome measures or (3) they were not peer-reviewed. The last searches were conducted on 3 April 2025.

23 studies with 540 PLwP met the inclusion criteria; 3 studies^30^,^32^ used RCT design, 2 studies^33 34^ included a control condition and 18 studies^812 35^,^50^ used non-controlled, pretest and post-test design.

The majority of included studies were conducted in English-speaking countries (Australia, Canada, USA), while two studies were from Asia^36 41^ and one from the Middle East.^32^ Only two studies used individual singing interventions,^32 41^ while 21 studies implemented group singing approaches. Further, five studies delivered online singing interventions using smartphone apps^32 36^ or online platforms.^45 48 49^ Most included studies involved a small number of participants ranging between 4^42^ and 28,^12 49^ except Tamplin *et al*’s^34^ and Irons *et al*’s^8^ studies that included 75 and 95 participants, respectively. In relation to study length, most participants received short-term to medium-term singing interventions ranging from 2 weeks^41^ to 6 months^8^ with two exceptions: Tamplin *et al*’s study^34^ lasted over 1 year and Evans *et al*’s study^39^ lasted 2 years.

### Description of singing intervention practices

All included studies’ rationale was built on the common mechanisms of speech and singing, where both speaking and singing require fine-tuned coordination of the respiratory and laryngeal function as well as neural networks. Such rationale encouraged a multidisciplinary approach to singing interventions, and both speech and language therapists and music therapists facilitated the singing interventions. In some cases, the researchers were trained in both professions,^12 37^ who combined speech therapy with choral singing. In other studies,^34 35 39 43 46 49^ both professionals cofacilitated sessions to optimise the cross-disciplinary benefits. The approaches taken by music therapists^35 38 41 44 45 47 48 50^ were also very similar to those of speech and language therapists, where the diaphragmatic breathing method, projecting one’s voice and expanding pitch ranges were the key components of singing intervention. The majority^833 34 38^,^42 44 47 48 50^ of singing interventions included singing well-known or familiar songs. Sessions typically lasted for 1 hour; several studies^12 31 32 45 47 49^ encouraged participants to adopt home practice. Details of the practices are provided in online supplemental table 1.

### Quality assessment of included studies

Modified Downs and Black checklist^23^ was used to assess three RCTs^30^,^32^ and two controlled studies.^33 34^ The RCTs were unable to blind participants, which is an inherent issue in singing studies; however, blinding assessors was implemented in Brooks *et al*,^33^ Butala *et al*^31^ and Mohseni *et al*.^32^ Only Mohseni *et al*^32^ performed a power calculation to determine a minimally required sample size. We also found inconsistencies between the trial protocol and the published paper in relation to reporting outcomes in Butala *et al*’s study.^31^ Additionally, we were uncertain about participants’ compliance with interventions in Mohseni *et al*’s study,^32^ as no details were reported. Brooks *et al*,^33^ Brown and Stegemöller^30^ Butala *et al*^31^ and Tamplin *et al*^34^ were rated as ‘fair’ indicating that there are some biases in relation to blinding assessor, sample size calculations and inconsistent outcome reports, while Mohseni *et al*^32^ were rated as ‘good’ indicating that there was less risk of bias regarding blinding participants and no details on compliance of intervention (online supplemental table 2A).

The remaining 18 pretest and post-test studies with no control groups are assessed using the NHLBI Quality Assessment Tool.^24^ Except for Irons *et al*’s study^8^ (n=95), the studies’ sample sizes were small, that is, between n=4 (Haneish)^42^ and n=28 (Tamplin *et al*^49^; Tanner *et al*)^12^). Other concerns include generally not having an interrupted time-series design where outcome measures were taken multiple times (NHLBI question #11), non-blinded assessors (NHLBI question #8) and having a significant loss to follow-up (>20%; NHLBI question #9) in Evans *et al*’s study,^39^ but follow-up was at 2 years. Additionally, most follow-up periods were short, that is, immediately postintervention. The quality assessment results are summarised in online supplemental table 2B.

### Meta-analyses

We conducted the following meta-analysis on primary and secondary outcomes: Two cross-over RCTs (Brown and Stegemöller^30^ Butala *et al*^31^) compared group singing with discussion (Butala *et al*^31^) and an expiratory training condition (Brown and Stegemöller^30^). From those two studies, we extracted the data from the baseline till the end of the first intervention period (ie, before the cross-over took place). Mohseni *et al*^32^ implemented a three-arm RCT involving (1) group singing combined with the conventional speech and language therapy, (2) speech therapy alone and (3) group singing alone. We extracted the data from group singing combined with conventional speech and language therapy and speech along group. The study by Tamplin *et al*^34^ offered two options to participants, to join a singing group or a control group. Within both the singing and control conditions, participants again selected to be in either the weekly or monthly group. For the meta-analysis, we only extracted data from the weekly singing and control groups, to increase similarity to other studies. Evans *et al*’s^39^ study data were not included in the meta-analysis due to the 2-year intervention period, which was much longer than other studies’ duration. Further, data from VHI was included in the QoL meta-analysis, as VHI is highly correlated with V-RQoL, and both instruments purport to measure the same construct.^51^ Further, there was no indication of reporting bias for any outcomes due to selective reporting.

#### Primary outcomes

##### Quality of life

First, we conducted a meta-analysis on RCT data: Three studies^31 32 34^ involving 79 participants assessed V-RQoL. The result revealed that the effects of singing interventions did not differ from comparators (SMD=0.59, 95% CI –1.35 to 2.53, I^2^=71.58%) with no publication bias. Moreover, due to high risk of bias (eg, not being able to blind participants to the intervention), inconsistency (based on high I^2^ value), imprecision (because the optimal information size was not met) and indirectness (due to varied control conditions across three studies), the certainty of the evidence was graded as very low (online supplemental table 3). This means that we currently have very little confidence in the effect estimate. The true effect is likely to be substantially different from the estimate of the effect.

Second, we conducted a meta-analysis on V-RQoL using data from non-RCTs and RCTs, where 10 studies^31^,^3436 38 41 46^ were included involving 127 participants. The result also revealed that V-RQoL did not change over time (SMD=0.16, 95% CI –0.02 to 0.34, I^2^=0%), and there was also an indication of publication bias. Begg and Mazumdar’s rank correlation test^29^ indicated the presence of publication bias (Z=2.24; p=0.03), and this was confirmed by Egger’s test (intercept=3.67, t=1.41, p=0.20). Imputed data points (two on the left) using the trim-and-fill method decreased the effect size, SMD=0.07, 95 % CI (−0.13 to 0.28) but did not alter heterogeneity (I^2^=10.79%).

Four studies^8 31 45 49^ used Parkinson’s specific measures of QOL, the PDQ-39^8 31 49^ and PDQ-8,^45^ but we were unable to carry out a meta-analysis due to an insufficient number of studies providing usable and similar data: Butala *et al*’s^31^ and Tamplin *et al*’s^49^ PDQ-39 data were not usable and scores from PDQ-39 and PDQ-8^45^ cannot be combined.

##### Well-being

For well-being assessments, no meta-analysis was conducted, as no study included psychometric measures.

### Secondary outcomes

#### Voice acoustic outcomes

##### Maximum phonation time

This outcome measures how many seconds one can hold a note on one breath, as an indicator of one’s breath control and laryngeal function.^52^

No meta-analysis was performed on controlled studies, as only one controlled study^33^ reported this outcome. One controlled study^33^ and 10 non-controlled studies^1234^,^36 40^ with a total of 157 participants were included in the meta-analysis. The result indicated a small but significant improvement from preintervention to postintervention (SMD=0.38, 95% CI 0.18 to 0.59, I^2^=23.48%); no publication bias was detected (online supplemental table 4).

##### Loudness of sustained vowel

This outcome evaluates loudness of voice during a sustained vowel production, measured in dB. First, meta-analysis of three controlled studies (one RCT^32^ and two non-RCTs^33 34^) involving 72 participants revealed effects in favour of singing compared with the comparator (SMD=0.67, 95% CI 0.29 to 1.05, I^2^=0%). There was no indication of publication bias. However, the certainty of this evidence was graded as very low, as there was a high risk of bias (due to being unable to blind participants to the intervention), imprecision (because the optimal information size was not met) and indirectness (due to varied control conditions across three studies) (online supplemental table 3). This means that we currently have very little confidence in the effect estimate. The true effect is likely to be substantially different from the estimate of the effect.

Second, turning to pretest and post-test non-controlled studies^32^,^3436 40 47 48^ involving 99 participants, meta-analysis revealed that there was a significant improvement from preintervention to postintervention: SMD=0.50, 95% CI 0.14 to 0.86, I^2^=50.96%. There was no indication of publication bias. Good *et al*’s study^40^ reported two different singing groups; therefore, we included data from both groups in the analysis (online supplemental table 4).

##### Loudness of reading

This outcome evaluates one’s vocal loudness during a reading task, measured in dB. No meta-analysis on RCTs was performed due to only two studies^31 32^ reporting this outcome. Meta-analysis of eight pretest and post-test studies^1236 38 42 46 48^,^50^ and two controlled studies^31 32^ with 143 participants revealed that there was no significant change from preintervention to postintervention (SMD=0.40, 95% CI –0.02 to 0.81, I^2^=69.92%); no publication bias was detected (online supplemental table 4).

##### Loudness of monologue

For the vocal loudness (dB) of monologue outcome, participants were asked to talk about their last holiday or describe a picture.

First, a meta-analysis of two RCTs^31 32^ and one controlled study^34^ with 79 participants indicated that the effects of singing interventions did not differ from comparators (SMD=0.48, 95% CI –0.17 to 1.13, I^2^=0%); there was no publication bias. And due to a high risk of bias (eg, not being able to blind participants to the intervention), imprecision (because the optimal information size was not met)—and indirectness (due to varied control conditions across three studies), the certainty of the evidence was graded as very low (online supplemental table 3). This means that we currently have very little confidence in the effect estimate. The true effect is likely to be substantially different from the estimate of the effect.

Second, we conducted a meta-analysis of seven studies including three controlled studies^31 32 34^ and four non-controlled studies^12 36 49 50^ involving 115 participants. The result revealed that there was no significant change from preintervention to postintervention (SMD=0.22, 95% CI –0.29 to 0.74, I^2^=78.44%) with no publication bias (online supplemental table 4).

##### Pitch range

This outcome is measured in semitones during a reading or conversational task.^53^ A meta-analysis of four non-controlled studies^12 42 47 48^ and one RCT^32^ involving 91 participants revealed that there was no significant change from preintervention to postintervention (SMD=0.57, 95% CI –0.01 to 1.15, I^2^=61.99%). There was limited evidence of publication bias, so trim and fill was executed and imputed data points (one on the left) decreased the effect size (adjusted SMD=0.48, 95% CI –0.11 to 1.08) (online supplemental tabe 4).

### Respiratory function

#### Maximal inspiratory pressure

This outcome is a measure of the strength of inspiratory muscles, primarily the diaphragm, measured in cmH_2_O. Three uncontrolled studies^37 47 48^ and one controlled study^34^ involving 65 participants evaluated the effect of group singing on MIP. There was a significant improvement from preintervention to postintervention (SMD=0.46, 95% CI 0.07 to 0.85, I^2^=0%) with no publication bias (online supplemental table 4).

#### Maximal expiratory pressure

This outcome is a measure of maximal strength of respiratory muscles, measured in cmH_2_O. Three uncontrolled studies^37 47 48^ and one controlled study^34^ involving 65 participants evaluated the effect of group singing on MEP. There was no improvement from preintervention to postintervention (SMD=0.58, 95% CI –0.25 to 1.41, I^2^=68.09%) with no publication bias (online supplemental table 4).

### Mental health

#### Depression

Questionnaires, such as Geriatric Depression Scale, DASS, Beck Depression Inventory and Montgomery Asberg Depression Scale, were used to assess depression in the included studies.

Five uncontrolled studies^8 38 41 45 49^ and two controlled studies^30 34^ involving 163 participants evaluated depression before and after the singing intervention, where reduced scores indicate improvements. There was no significant improvement in depression from preintervention to postintervention (SMD= –0.16, 95% CI –0.42 to 0.01), I^2^=35.71%). There was no publication bias (online supplemental table 4).

#### Anxiety

Questionnaires, such as DASS, were used to assess anxiety in the included studies.

Two uncontrolled studies^8 49^ and two controlled studies^30 34^ involving 128 participants evaluated anxiety before and after the singing interventions, where reduced value indicated an improvement. There was no significant improvement from preintervention to postintervention (SMD= –0.05, 95% CI –0.57 to 0.48, I^2^=69.02%) with no publication bias (online supplemental table 4).

#### Stress

Questionnaires, such as the DASS, were used to assess stress in the included studies.

Two uncontrolled studies^8 49^ and two controlled studies^30 34^ involving 121 participants assessed anxiety preintervention and postintervention. A meta-analysis revealed that there was no significant improvement from preintervention to postintervention (SMD=–0.13, 95% CI –0.75 to 0.50, I^2^=73.92%) with no publication bias (online supplemental table 4).

### Motor function measures

No meta-analysis was possible due to a small number of studies^46 49^ reporting these outcome measures using different measures and times.

### Adverse effects

There were no adverse effects reported in any study.

### Follow-up

Meta-analysis was not possible due to only two studies^32 41^ reporting follow-up data, and the periods varied between 3^32^ and 6^41^ months.

## Discussion

### Summary of main results

The aim of this systematic review was to determine the effects of singing interventions for health outcome measures in PLwPs. We focused primarily on QoL and mental health, and, secondarily, on voice, respiratory and motor functions. Following the comprehensive searches, 23 singing studies with a total of 540 participants were included in subsequent analyses. These studies varied in terms of their design: 3 RCTs,^30^,^32^ 2 studies with a control group^33 34^ and 18 non-controlled, preintervention and postintervention studies (online supplemental table 1).

Meta-analyses of controlled studies indicated that singing interventions may be effective for improving voice loudness of sustained vowel, but not effective for V-RQoL and voice loudness of monologue. But the certainty of this evidence was very low due to a high risk of bias, inconsistency, indirectness and imprecision. For the other outcome measures (mental health, respiratory and motor functions), there was insufficient studies for meta-analysis.

Additionally, meta-analyses including data from both controlled and uncontrolled studies indicated that there were improvements from preintervention to postintervention on three outcome measures (maximum phonation time, vocal loudness of sustained vowel and maximum inspiratory pressure) (online supplemental table 4). It is worth stressing that these meta-analyses were limited to preintervention and postintervention studies due to insufficient numbers of controlled studies to perform meta-analysis. Thus, given the insufficient evidence, we cannot determine the effects of singing interventions for PLwPs.

As discussed in a previous paper^54^ on methodological weaknesses in singing studies, our review also identified a number of methodological limitations in the included studies: most studies’ methods were not rated highly due to small sample sizes, blinding of participants and assessors, and weak study design; examination of moderation using study quality was not possible due to the variety of study designs. Indeed, there were only three RCTs, and their sample sizes ranged between 14 (in Brown and Stegemöller’s study)^30^ and 26 (in Butala *et al*’s study)^31^; the evidence from these studies is equivocal. Studies also varied considerably in the assessments made with only MPT and reading loudness being assessed in more than half of the studies; other communication, respiratory, mental health and QoL indices were assessed in between 20% and 40% of studies. This may have been due to the different settings and countries where the studies were conducted.

Parkinson’s is a progressive condition, which is another challenge for singing interventions to demonstrate any significant benefits over time. It is questionable whether Parkinson’s specific QoL questionnaires are suitable for singing interventions. For example, the most used QoL questionnaire, PDQ-39 or PDQ-8, assesses the difficulties and severities of Parkinson’s from medical perspectives. Singing studies may not be able to capture the QoL-related improvements,^9^ such as experiencing positive emotions, increased motivations and having purposes, through these measures. Taken together, there is currently no convincing evidence for the benefits of singing for PLwP, but there are some vocal and other outcomes that could be targets for more robust studies of better quality.

In this systematic review, we also examined the singing practices used in the included studies. Across the 23 included studies, there are commonalities (online supplemental figure 2): singing sessions started and closed with conversations or ‘check-ins’ with participants; some physical warm-ups were then introduced, including music-guided relaxation,^35^ stretching, trunk rotation^31^ and oro-facial-neck-shoulders muscular relaxation.^37^ Building on physical warm-ups, singers engaged with breathing exercises with a focus on fully involving the diaphragm, which enables the singer to engage the lungs fully. The included studies^832 36^,^47 49 50^ emphasised deep breathing, abdominal breathing exercises and practised them for 5–10 min. Vocal warm-ups were one of the most important elements in singing practice reported in the included studies. Studies^34 47^ introduced targeted activities, such as lip buzzing, glissandos and messa di voce, to impact on the laryngeal mechanisms that are affected by Parkinson’s. Two studies by Tamplin *et al*^34 49^ reported ‘high-intensity vocal exercises’; however, they did not provide further details. Similarly, Azekawa and Lagasse^35^ reported ‘therapeutic singing’ without details. Researchers^12 37^ who have qualifications as both classical singers and speech and language therapists reported including ‘sustained, loud vowel sounds at various pitches, pitch glides, repetition of common phrases and melodious practice of vocal music’. Lip trilling^55^ and sirening^56^ exercises were also emphasised, for example, in Evans *et al*’s study.^39^ Lip trilling is recommended as a beneficial exercise for the lips, oral cavity and vocal fold, which are involved in making sounds. Lip trilling can also support improving breath control, as well as improving the pitch range of the voice.^57^ Additionally, Butala *et al*’s study^31^ adopted Estill Voice Training principles and exercises from the Kodály and Orff methods: Estill Voice Training principles emphasise understanding the healthy vocal function to maximise voice control for speaking, singing and overall health.^58^ Kodály methods use hand signs for vocal/choral training, while Orff methods use body movement, speech and percussion instruments to make music.^59^ In summary, the vocal warm-ups were an important element in singing studies, carried out for between 15 and 30 min, with the aim to improve voice functions in PLwP, who present unclear and often quite breathy voice and slurred speech due to difficulties with movements of muscles required for speaking.^60^

Following the vocal warm-ups, singing songs was offered with the aim of putting all the breathing and vocal techniques into songs. There was variety in the song repertoire including: familiar songs (eg, in Brooks *et al*’s study^33^; songs from musicals (eg, in Higgins and Richardson’s study^43^; well-known traditional songs (eg, in studies by Chan *et al*,^36^ Evans *et al*,^39^ Tamplin *et al*^34^); choral pieces including liturgical chants^37^; participants’ preferred or chosen songs^8 38 42 50^ and popular songs.^46 47^ Most studies reported singing for 20–30 min at a single session (online supplemental table 1).

In summary, the current systematic review identified breathing exercises and voice warm-ups as key elements of singing interventions (online supplemental figure 2). Researchers also regarded singing popular or familiar songs, or participants’ chosen songs; this is important to maximise the effort and enjoyment during singing. were facilitated by either music therapists or professional musicians and in some cases with speech therapists’ input or co-facilitation (eg, in Tamplin *et al*’s studies).^34 49^ Online supplemental figure 3 presents facilitators’ professional backgrounds in the singing studies: singing interventions were facilitated most frequently by music therapists, followed by professional musicians combined with speech therapists’ input, and choral director/professional musicians.

### Implications for research

More evidence from RCTs on the effects of singing for PLwP is needed because singing has become increasingly popular and been advocated as a non-pharmacological intervention with a range of potential benefits. Thus, future work should address the following:

1. Rigorous and well-conducted RCTs are needed. Trials should be sufficiently powered and include appropriate comparisons; comparing singing with an active comparator is desirable.
2. Longer-term follow-up is needed to establish the long-term benefits of singing. For people who enjoy group singing, it is part of their routine activities and something that they look forward to. However, in this systematic review, we identified only two studies^32 41^ that reported follow-up data.
3. Future trials should use more consistent and objective outcome measures. In terms of voice and communication-related measures, we would recommend including max phonation time and loudness assessments using recommended guidelines,^61^ as well as other aspects of communication that are important to PLwP and their carers. Additionally, there are smartphone apps and other technology providing voice assessments in a convenient way. This systematic review also found that QoL and respiratory function assessments are frequently included in the singing studies. Further, mental health is another potential indicator of benefits from singing interventions that could be assessed in future studies. Finally, swallowing function^62^ and motor function^63^ could be investigated.
4. Given the difficulty of blinding participants in singing studies, trials should ensure that people involved with data collection and analysis are blinded to condition to reduce bias.
5. Trials should include a very clear description of the singing intervention, that is, the contents of singing sessions and delivery modes, as it is not yet known what an effective singing intervention should entail in relation to vocal exercises, song singing and optimal delivery format including frequency, number and duration of sessions.
6. Future trials should consider the cost-effectiveness of singing interventions. To date, no study has evaluated the cost-effectiveness of singing interventions for people with Parkinson’s. And this information may guide healthcare providers about its inclusion within care pathways for PLwP.
7. In relation to singing practice, we recommend including breathing exercises, vocal warm-ups and participants’ preferred song singing in each session, as these elements were found to be important in the singing studies.
8. Additionally, singing facilitators may need to learn specific knowledge and skills to work with PLwP, which is currently not part of the curriculum at music or conservatoire specialised training. Ongoing support for singing facilitators may also be needed. Moreover, singing facilitators should consider collaborating with local multidisciplinary Parkinson’s rehabilitation teams, so that singing facilitators could provide appropriate and high-quality singing sessions.

### Limitations and strengths

This systematic review presents the most up to date evidence of singing interventions for PLwP and summarises the singing practices used in the interventions. We identified 18 pretest/post-test non-controlled studies; only five studies had control conditions. This is the current landscape of evidence on the effects of singing interventions for PLwP. Accordingly, evidence is weak and limited. Relatedly, the quality of the studies was limited due to a high risk of bias, inconsistency, imprecision and indirectness. Very few follow-up data were presented; only two studies included follow-up data. Further, outcome measures were collected inconsistently from study to study; methodological details were sparse.

## Conclusions

The current systematic review and meta-analysis involving a total of 23 singing studies with 540 PLwP found that there were no effects of singing interventions for QoL, respiratory function, mental health and motor function; however, there were some indications of effects of singing for some of vocal loudness outcomes based on three controlled studies. Evidence of other outcomes was more limited by pre–post study designs. The meta-analyses results provide estimates for the future larger controlled trials. This systematic review also underlined the core elements of current singing practice and recommended including breathing exercises, vocal warm-ups and singing preferred songs. Further studies with robust methods are required to inform both healthcare professionals and PLwPs on the effects of singing.

## Supplementary material

10.1136/bmjopen-2024-089154 online supplemental file 1

## Data Availability

All data relevant to the study are included in the article or uploaded as supplementary information.
