# Supplementary material for: Singing interventions for people living with Parkinson’s: a systematic review and meta-analysis
Source: BMJ Open. 2025 Nov 24;15(11):e089154. doi: 10.1136/bmjopen-2024-089154 (PMC12645629; doi:10.1136/bmjopen-2024-089154)

**Supplementary files**

| **1. Searches (last search performed on 3/04/2025)** |
| --- |

| **Database** | **Search strategies** |
| --- | --- |
| **PsycInfo** (EBSCO)  888 hits | 1 exp Parkinsons Disease/  2 parkinson*.mp  3 1 OR 2  4 singing/  5 (sing OR singing OR singer*).mp  6 vocalization/  7 voice/  8 ((voice* or vocal*) adj3 (exercis* or train* or intervention* or rehabilitat* or program* or therap* or treatment*)).mp  9 (choir* OR choral* OR chorus*).mp  10 exp music therapy/  11 music/  12 music*.mp  13 or/4-12  14 3 AND 13  **Limiters** - Publication Year: 1999-2025  **Expanders** - Apply equivalent subjects  **Search modes** - Proximity |
| **Medline (**EBSCO)  886 hits | 1 exp Parkinsonian Disorders/  2 parkinson*.mp  3 1 OR 2   4 singing/  5 (sing OR singing OR singer*).mp     6 ((voice* or vocal*) adj3 (exercis* or train* or intervention* or rehabilitat* or program* or therap* or treatment*)).mp  7 (choir* OR choral* OR chorus*).mp  8 music therapy/  9  music/  10 music*.tw, kf  11 or/4-10  12 3 AND 11  **Limiters** - Publication Year: 1999-2025  **Expanders** - Apply equivalent subjects  **Search modes** - Proximity |
| **CINAHL** (EBSCO)  804 hits | S1 MH “Parkinsonian Disorders+”  S2 parkinson*  S3 S1 OR S2  S4 MH “singing”  S5 sing OR singing OR singer*  S6 (voice* or vocal*) N3 (exercis* or train* or intervention* or rehabilitat* or program* or therap* or treatment*)  S7 choir* OR choral* OR chorus*  S8 MH music therapy  S9 MH music  S10 music*  S11 S4 OR S5 OR S6 OR S7 OR S8 OR S9 OR S10  S12 S3 AND S11  **Limiters** - Publication Year: 1999-2025  **Expanders** - Apply equivalent subjects  **Search modes** - Proximity |
| **Web of Science Core Collection**  657 hits | 1 TS=(parkinson*)  2 TS=(sing*)  3 TS=((voice* OR vocal*) NEAR/3 (exercise*))  4 TS=((voice* OR vocal*) NEAR/3 (train*))  5 TS=((voice* OR vocal*) NEAR/3 (intervention*))  6 TS=((voice* OR vocal*) NEAR/3 (rehabilit*))  7 TS=((voice* OR vocal*) NEAR/3 (program*))  8 TS=((voice* OR vocal*) NEAR/3 (therap*))  9 TS=((voice* OR vocal*) NEAR/3 (treatment*))  10 #3 OR #4 OR #5 OR #6 OR #7 OR #8 OR #9  11 TS=(choir*)  12 TS=(choral*)  13 TS=(chorus*)  14 TS=(music*)  15 #11 OR #12 OR #13 OR #14  16 #15 OR #10 OR #2  17 #16 AND #1  **Limiters** - Publication Year: 1999-2025; Article (Document Types); Clinical Neurology or Neurosciences (Web of Science Categories), Citation Topics (Neurodegenerative Diseases; Neuroscience; Parkinson's Disease) and Human |
| **Google Scholar** | (Parkinson’s OR Parkinsonian Disorders) AND (singing OR sing OR choral OR chorus)  Limited to the first 100 articles. |
| **Other sources: Citation searches** | 11 |

| **List of studies that were excluded** |
| --- |

1. **Inappropriate intervention (e.g., not singing, singing interventions less than two weeks) (n=12)**

- Satoh, M., & Kuzuhara, S. (2008). Training in mental singing while walking improves gait disturbance in Parkinson's disease patients. European neurology, 60(5), 237–243.
- Kim, Y., Sidtis, D., & Sidtis, J. J. (2023). Singing and Speaking Ability in Parkinson's Disease and Spinocerebellar Ataxia. Journal of speech, language, and hearing research: JSLHR, 66(1), 126–153.
- Lee, S. J., Dvorak, A. L., & Manternach, J. N. (2024). Therapeutic Singing and Semi-Occluded Vocal Tract Exercises for Individuals with Parkinson's Disease: A Randomized Controlled Trial of a Single Session Intervention. Journal of music therapy, 61(2), 132–167.
- Searl, J., Wilson, K., Haring, K., Dietsch, A., Lyons, K., & Pahwa, R. (2011). Feasibility of group voice therapy for individuals with Parkinson's disease. Journal of communication disorders, 44(6), 719–732.
- Pacchetti C, Mancini F, et al. Active music therapy in Parkinson's disease: an integrative method for motor and emotional rehabilitation. Psychosom. Med. 2000;62(3):386-393.
- Stegemöller EL, Zaman A, Shelley M, Patel B, Kouzi AE and Shirtcliff EA (2021) The Effects of Group Therapeutic Singing on Cortisol and Motor Symptoms in Persons With Parkinson’s Disease. Front. Hum. Neurosci. 15:703382.
- Stegemoller E, Forsyth E, Patel B, et al. Group therapeutic singing improves clinical motor scores in persons with Parkinson’s disease. BMJ Neurology Open 2022;4:e000286.
- Harrison EC, McNeely ME, et al. The feasibility of singing to improve gait in Parkinson disease. Gait Posture 2017;53:224-229
- Harrison EC, Horin AP, et al. Internal cueing improves gait more than external cueing in healthy adults and people with Parkinson disease. Sci. Rep. 2018;8(1):15525.
- Harrison EC, Horin AP, et al. Mental singing reduces gait variability more than music listening for healthy older adults and people with Parkinson Disease. J Neurol Phys Ther 2019;43(4):204–211
- Harris, R., Leenders, K. L., & de Jong, B. M. (2016). Speech dysprosody but no music 'dysprosody' in Parkinson's disease. Brain and language, 163, 1–9.
- Liao YH, Lin TY, et al. Can occupational therapy manpower be replaced with social robots in a singing group during COVID-19? Work (Reading, Mass.) 2021;68(1):21–26.

1. **Inappropriate outcome measures (e.g., qualitative, other outcomes than inclusion criteria, systematic reviews) (n=16)**

- Baird AD, Abell R, et al. Group singing enhances positive affect in people with Parkinson’s disease. Music and Medicine 2018;10(1):13–17
- Gaspar, Phyllis M.Gaspar, Phyllis M. et al.(2020). Effects of Participation in Tremble Clefs by Individuals with Parkinson’s Disease on Voice-Related Quality of Life. Journal of the American Medical Directors Association, 21:3, B19 - B20
- Rong, P., & Benson, J. (2023). Intergenerational choral singing to improve communication outcomes in Parkinson's disease: Development of a theoretical framework and an integ
- rated measurement tool. International journal of speech-language pathology, 25(5), 722–745.
- Yeo, M. S., Hwang, J., Lee, H. K., Kim, S. J., & Cho, S. R. (2024). Therapeutic singing-induced swallowing exercise for dysphagia in advanced-stage Parkinson's disease. Frontiers in neurology, 15, 1323703.
- Pohl, P., Wressle, E., Lundin, F., Enthoven, P., & Dizdar, N. (2020). Group-based music intervention in Parkinson's disease - findings from a mixed-methods study. Clinical rehabilitation, 34(4), 533–544.
- Abell, R. V., Baird, A. D., & Chalmers, K. A. (2017). Group singing and health-related quality of life in Parkinson’s disease. Health Psychology, 36(1), 55–64.
- Fogg-Rogers L, Buetow S, Talmage A, et al. Choral singing therapy following stroke or Parkinson’s disease: an exploration of participants’ experiences. Disabil Rehabil. 2016;38:952–962.
- Hersh, D., Kong, S. J., & Smith, J. (2023). It's quite good fun: A qualitative study of a singing/songwriting programme for people with Parkinson's disease and their spouses. International journal of language & communication disorders, 58(6), 2103–2116.
- Stegemöller EL, Hibbing P, et al. Therapeutic singing as an early intervention for swallowing in persons with Parkinson's disease. Complement Ther Med 2017;31:127-133
- Elefant C, Lotan M, et al. Effects of music therapy on facial expression of individuals with Parkinson's disease: A pilot study. Musicae Scientiae 2012;16(3):392-400.
- Segall LE. The effect of group singing on the voice and swallow function of healthy, sedentary, older adults: A pilot study. The Arts in Psychotherapy 2017;55:40-45.
- Barnish MS, Barran SM. A systematic review of active group-based dance, singing, music therapy and theatrical interventions for quality of life, functional communication, speech, motor function and cognitive status in people with Parkinson's disease. *BMC Neurol.* 2020;20:371 doi: 10.1186/s12883-020-01938-3
- Irons JY. Systematic review of the effects of singing for health outcomes in people living with Parkinson’s. [review protocol registration] 2024: Retrieved from osf.io/nrjea
- Ang K, Maddocks M, *et al.* The Effectiveness of Singing or Playing a Wind Instrument in Improving Respiratory Function in Patients with Long-Term Neurological Conditions: A Systematic Review. *J Music Ther.* 2017;54:108-131.
- Sotomayor MJM, Arufe-Giraldez V, *et al.* Music Therapy and Parkinson's Disease: A Systematic Review from 2015-2020. *Int J Environ Res Public Healt* 2021;18:11618
- Fancourt D, Finn S. What is the evidence on the role of the arts in improving health and well-being? A scoping review. WHO Health Evidence Network Synthesis report 67 2019. ISBN: 978 92 890 5455 3
- Monroe P, Halaki M, *et al.* The effects of choral singing on communication impairments in acquired brain injury: A systematic review. *Int J Lang. Commun Disord* 2020;55:303-319.

1. **Non-peer-reviewed materials (n=2)**

- Matthews RM. Acoustic, respiratory, cognitive and wellbeing comparisons of two groups of people with Parkinson’s disease participating in voice and choral singing group therapy (VCST) versus a music appreciation activity. PhD Thesis 2018;The University of Auckland, NZ.
- Wünnenberg, E, Jungen, M. Singing – tune into self-regulation: A holistic system perspective [abstract]. *Mov Disord.* 2019; 34 (suppl 2).

| **Table 1. Characteristics of the Included Studies (k=23, n=540)** |
| --- |

| **Study ID:**  **First author, (year),**  **Country**  **(reference#)** | **Aim & approach** | **Study design & participants & setting** | **Singing intervention** | **Outcome measures** | **Results** |
| --- | --- | --- | --- | --- | --- |
| 1. Azekawa (2018)   USA  #35 | - To investigate the feasibility of a group singing/music therapy protocol for treating voice and speech deficits  - Music Therapy approach | - Pre- & post-test design without a control group  - n=5 (3 males); *M*age=70.8 yrs (SD=10.30); Time since Dx=8.2 yrs (range 1-15 yrs); H&Y stage=2.5 (range 2.5-3)  - University music therapy lab setting  - A 50-min weekly group singing for 6 sessions (6 weeks)  - led by a music therapist | - Group singing music therapy protocol  - Opening exercise (5 min); Vocal intonation therapy (15 min); Therapeutic singing (10 min); Break (5 min); Therapeutic singing (10 min); Closing (5 min: music guided stretching and relaxation)  - A speech language therapist was present  to model each vocal or singing exercise movement | - Voice: loudness of sustained vowel phonation & reading; diadochokinetic test; speech intelligibility  - Acoustic analyses using PRAAT | - All assessments were tolerated & acceptable.  - 71% of participants completed; 29% dropped out due to medical reasons.  - Participants’ experience of the intervention was positive. |
| 1. Brooks (2021)   USA  #33 | - To examine the effect of a group therapeutic singing intervention on voice, cough, and quality of life  - Speech and language rehabilitation approach | - Repeated measures design with a non-randomised control group (usual care)  - n=19 (Singing gr: n=10, *median*= 68 yrs, 10 males); (Control gr: n=9, median=69 yrs, 5 males);  - in a community-based outpatient setting  - led by a music therapist | - 12-week weekly group singing;  - high intensity vocal exercise incl. lip buzzing/ lip trills; glissandos; Mezze de voce; articulation exercises (30 min);  - singing familiar songs (15 - 20 min) | - Voice measure: vowel duration, intensity;  - Speech measure: using perceptual rating on breathiness, hoarseness, pitch level, intelligibility;  - Cough measure: Peak Expiratory Flow;  - Quality of Life measure: VHI & Communicative Effectiveness Survey-revised (CES-R) | A marked improvement, although statistically not significant, in peak expiratory flow during cough was observed in the singing group. This may indicate that singing has potential benefits for increasing laryngeal and respiratory muscle strength, thus delaying the impending swallowing and respiratory complications due to progressive nature of Parkinson’s. |
| 1. Brown   (2024)  USA  #30 | - To investigate and compare the effects of Expiratory Muscle Strength Training and Therapeutic Singing on QOL, depression, and anxiety  - Music Therapy approach | - Randomised cross-over design & mixed method  - n=14 (Singing first gr: n=7, *M*age= 69 yrs (SD=7), 3 males, Time since Dx=9 ± 7 yrs); (EMST gr: n=7, *M*age= 70 yrs (SD=7), 4 males, Time since Dx=8 ± 8 yrs);  - in a community-based outpatient setting  - led by a music therapist | - Singing: individual home-based singing  using pre-recorded videos via a private YouTube channel; 5 x per wk for 4 wks  - Control intervention: using EMST 150 device 5 x per wk, 5 sets of 5 breaths for 4 wks  - led by music therapist | - Quality of Life measure: PDQ-39  - Mental health: GDS, PAS  - Survey after treatment: measure of acceptability, 6 questions, 5-point Likert scale  - Daily diary log: measure adherence  - 3 Assessments: at baseline, 4 wks (before cross-over), 4 wks post-intervention | - Participants reported breath strength and vocal volume after both interventions |
| 1. Butala   (2022)  USA  #31 | - To examine the effects of group singing programme for QoL, self-efficacy, mood, and voice/speech function  - Neuro-Rehabilitation approach | - Randomised, single (assessor)-blinded cross-over design  - n=26 (*M*age=68.6 yrs (range 55–89); 16 males; H&Y stage=2.3;  - Singing first group (n=13) for 12 wks, then cross over to the control group for 12 wks (discussion);  - Weekly 1.5-hr group singing  - led by professional choral director | - Estill Voice Training principles + exercises adopted from the Kodaly & Orff methods  - Solfege using Curwen’s hand signs;  - Warm-up (10 min) incl. stretching, sit to stand, and trunk rotation;  - Rhythmic learning;  - Repertoire incl. well-known/traditional songs  - Home practice was encouraged | - Voice: loudness during sustained vowel, reading & conversation  - QoL: PDQ-39; V-RQoL  - Parkinson’s disability: MDS-UPDRS;  - Assessments at baseline, 6, 12, 18, 24 and 30 weeks | - 81% completion rate was reported.  - Improvements over 24-wk in conversational speaking volumes, PDQ-39 Body Discomfort & Emotional Wellbeing domains were observed.  - There were a significant between group differences at baseline in age, gender distribution, disease severity & several voice measures. |
| 1. Chan   (2019) Malaysia  #36 | - To assess the feasibility of using smartphone videoconference to deliver intensive voice therapy  - Speech language rehabilitation approach | - Pre- & post-test design without a control group  - n= 11 (*M*age= 65 yrs, SD=6.9 yrs); 7 males;  - Twelve 30-min sessions delivered via smartphone videoconferencing over 4 weeks  - Speech Language Therapist undertook assessments  - NR: facilitator of singing programme | - Telehealth approach adopted from the LSVT and vocal function exercises  - Incl. vocal hygiene education, warm-up practices, breathing exercises, sustained vowel practice, voice gliding and a max. speech loudness task | - Voice: loudness during reading, monologues tasks & max. phonation time; VHI-10; Auditory-perceptual measurement (CAPE-V)  - 12-item smartphone-base therapy satisfaction questionnaire | - 100% attendance and completion were reported.  - Participants demonstrated louder voice at post-intervention.  - Overall, high satisfaction with smartphone videoconferencing delivery. |
| 1. Di Benedetto (2009)   Italy  #37 | - To assess a new voice rehabilitation programme (voice therapy + choral singing)  - Speech and language therapy + choral singing approach | - Pre & post-design comparing 1hr weekly singing with 2 hrs weekly singing  - n=20 *M*age=66 yrs (SD=9); 13 males; Time since Dx=7 yrs (SD=4.1); H&Y median stage=2  - led by speech language therapist with choral director experience  - for 13 weeks, at hospital chapel setting | - Combination of voice and choral singing treatment  - Speech therapy (2 sessions of 1 hr weekly, a total of 20 hrs) + Choral singing (2 hrs weekly session for 13 wks, a total of 26 hrs)  - Speech therapy incl. oro-facial-neck-shoulders muscular relaxation, respiratory/laryngeal/prosodic exercises  - Choral singing incl. adjusted/simplified rhythmic popular and liturgical chants | - Voice: Max phonation time; loudness; fundamental frequency; jitter, shimmer, peak amplitude carination & quality of voice;  - Respiratory function measures: MIP, MEP, FVC, FEV1, FRC, airway resistance | - Improvements were detected in max phonation time, prosodia & respiratory muscle strengths. |
| 1. Elefant   (2012)  Norway  #38 | - To assess the effects of group singing on voice/communication, singing, quality of life and depressive symptoms  - Music therapy approach | - Pre & post-design without a control group  - n=10 *M*age=64 (SD=8.9); 7 males; Time since Dx =4.2 (SD=1.4)  - At rehabilitation hospital setting  - Assessments at baseline, midway (10 wk) & post (20 wk)  - Once weekly for 20 wks;  - led by music therapist | - Group voice and singing intervention  - Introduction (5-10 min); Breathing exercises (5-10 min); Vocal exercises (5-10 min); Song exercises (participant-chosen songs; 20-30 min) | - Measures on spoken passage; voice range, song singing; VHI; Montgomery & Asberg Depression rating | - Improvements in singing ability was detected; slight improvements |
| 1. Evans   (2012)  UK  #39 | - To assess the benefits of group singing for voice/communication and quality of life  - Parkinson’s multi-disciplinary rehabilitation approach (Professional musician + Speech & Language therapist + Parkinson's nurse) | - Pre & post-design  - n=17 *M*age=67 yrs (range 48-81 yrs); 11 males; Time since Dx=9 yrs (range 2-21 yrs)); N=10 completed post-assessment at 2-year  -led by a professional musician in collaboration with a speech language therapist & Parkinson’s nurse  - Weekly sessions for 2 yrs | - Positive Posture, breathing exercises, vocal warm-ups (lip trilling, sirening);  - Call & response well-known songs | - QoL: PDQ-39;  - Voice: Frenchay Dysarthria assessment | - Improvements observed in Dribble, Laryngeal pitch, Laryngeal volume, and Laryngeal speech in Frenchay Dysarthria assessment. |
| 1. Good   (Canada)  2022  #40 | - To assess the impacts of singing in a community choir on vocal quality  - Singing for health approach + asset-based approach | - Pre- & post-test design without a control group  - n=22 (from 2 locations: gr A=Toronto N=12, (8 males) *M*age=69.58 yrs (SD=9), gr B=Winnipeg N=10 (5 males), *M*age=73.10 yrs (SD=5))  - Community choir  - led by professional choir director (gr A); music therapist (gr B) | - Group singing for 12-wk (research data taken from ongoing choir)  - 1 hr session consisting of warm-up & vocal exercises, song singing  - home practice was encouraged using song lyrics & audio tracks | - Voice: Loudness; Max. Phonation Time; pitch range; acoustic analysis (jitter, shimmer) | - Only Gr B improved vocal loudness; both group improved on Max. phonation time  - improvements on voice acoustic qualities (jitter & shimmer) indicating less harsh or coarse speaking voice |
| 1. Han   (2018)  South-Korea  #41 | - To assess vocal quality and depressive symptoms following an individual singing programme  -Music therapy approach | - Pre- & post-test design without a control group  - n=9; *M*age=65.7 (SD=7.7); T since Dx=4.8(3.0); H&Y stage=2 (range 1.5 to 3);  - 6 sessions over 2 weeks; follow-up at 6-month  - led by music therapist  - at an outpatient clinic | - Individual Therapeutic Singing Programme for Parkinson’s Disease (ITSP-PD)  - Exploration level (1^st^ & 2^nd^ session); Reinforcement level (3^rd^ & 4^th^); Integration level (5^th^ & 6^th^)  - Each session (50 min) incl. pre-interview & self-report (5 min); therapeutic vocalization (20 min); vocal improvisation & song-making activities (20 min); post-interview & self-report (5 min) | - Voice: Max. Phonation Time; VHI;  - QoL: V-RQoL  - Mental health: GDS | - Improvements were observed in MPT, VHI & depressive symptoms. |
| 1. Haneish   (2001)  USA  #42 | - To assess the benefits of a Music Therapy Voice Protocol for voice/ communication  - Music therapy approach | - Pre- & post-test design without a control group  - n=4 (females); age range 67-77 yrs;  - 12 sessions over 1 month  - led by music therapist | - Music Therapy Voice Protocol  - Opening conversation (3 min); Warm-up (5 min) incl. Abdominal breathing exercises; Vocal exercises (20 min); Singing exercises (15 min) incl. preferred songs; Max. Duration of sustained vowel phonation (5 min); Review & speech exercise (9 min); Closing conversation (3 min) | - Voice: speech Intelligibility inventory; reading Rainbow passage; conversation; pitch range;  - Mood: Feeling scale | - Improvements were observed in vocal loudness and mood. |
| 1. Higgins   (2019)  USA  #43 | - To explore the benefits of a 11-week choral singing intervention  - Speech and language rehabilitation approach | - Pre- & post-test design without a control group  - n=10 (5 males; *M*age=73 yrs (SD=6.65); Time since Dx=7.6 yrs (SD=2.46)  - 90-min weekly for 11-week  - led by a trained vocal performer + 2 speech & language specialists | - Choral singing protocol adopted from the MTVP by Haneishi (2001)  - Aimed to increase respiratory effort, vocal loudness, prosody, orofacial movement, and movement of the articulators  - Physical warm-up (5 min), breathing exercises (5 min), vocal warm-ups (5 min), opening conversation (5 min), singing (65 min; 5 musical pieces), closing conversation (5 min) | - Tongue excursion during vowel articulation: VSA (Vowel Space Area); Speech intelligibility | - Improvements in VSA and speech intelligibility at post-test were observed.  - 100% completion rate was reported. |
| 1. Irons   (2021)  UK,  South-Korea,  Australia  #8 | - To explore the benefits of group singing on QoL, mental health in participants in South-Korea, Australia and UK  - Singing for health approach | - Pre- & post-test design without a control group  - n=95 (43 males; *M*age= 70.26 yrs (SD= 8.36); Time since Dx=6.72 yrs  - led by trained singing for health practitioners  - 1hr weekly group sessions for 6-month | - *Sing to Beat Parkinson’s®* with a focus on abdominal breathing, movements while singing, projecting the voice, & expanding pitch.  - Welcome and hello song (5 min); breathing exercises (5 min); vocal warm-ups (5 min); warm-up songs (rounds, simple songs, 10 min); sharing time (e.g., birthdays, 5 min); participants’ preferred song singing (30 min); refreshment | - QoL: PDQ-39  - Mental health: DASS-21 | - Improvements in some domains of QoL (Social Support and Stigma), and mental health (Anxiety & Stress).  - Minimal clinically important differences of PDQ-39 were detected in the Stigma and Bodily Discomfort domains. |
| 1. Lewellen   (2020)  USA  #44 | - To examine the effects of participation in a therapeutic singing protocol on the voice quality and wellbeing  - Music therapy approach | - Pre- & post-test mixed-method design without a control group  - n=12 (10 males; *M*age= 66.5 yrs (range 60-86); Time since Dx=7.9 yrs  - led by music therapist  - 50-min weekly group choir sessions for 8 wks | - Therapeutic Singing Protocol (Yinger, 2012)  - Opening song and conversation (5 min); posture/body alignment (2 min); breathing exercises (3 min); voice warm-ups (5 min); applied technique (10 min); singing familiar songs (20 min); closing song & conversation (5 min) | - Voice: loudness (reading & Messa di Voce tasks) | - Exit interview: participants reported positive, enjoyable experiences; heightened sense of wellbeing  - primary gain of the intervention was associated with improvement in singing task (Messa di Voce) |
| 1. Mohseni (2023)   Iran  #32 | - To examine the effects of a new tele-rehabilitation programme, a combining of conventional speech therapy and singing intervention, on voice deficits  - Speech and language rehabilitation approach | - Three-armed, assessor-masked randomised controlled trial (singing + speech combined; speech only; singing only)  - n=33 (*M*age=58.88 yrs (SD=8.12); 25 males; Time since Dx= 7.15 yrs (SD=1.48); H&Y stage=1.85  - Combination therapy group co-facilitated by a speech and language therapist & professional singing teacher;  - Speech only group led by a speech and language therapist;  - Singing only intervention was delivered by a professional singing teacher. | -Individual 12 tele-rehabilitation sessions over 4 wks;  - Via video calls on a free WhatsApp Messenger;  - home exercises on non-treatment days  - Combination therapy group received breathing exercises; speech exercises & singing exercises  - Speech only group received speech therapy;  - Singing only group received singing | - Voice: loudness, jitter, shimmer, pitch range, max frequency range; VHI  -Assessments at baseline | - The combination therapy group (speech therapy + singing) demonstrated improvements in voice/ communication outcome measures. The gains were preserved at the 3-month follow-up.  - Tele-rehabilitation combination therapy might be an inexpensive and enjoyable behavioural treatment with several advantages, such as easy access, help with self-management. |
| 1. Shah-Zamora   (2024)  USA  #45 | - To evaluate the effect of virtual group music therapy singing on apathy  - To assess caregivers’ burden  - Music therapy approach | - Pre- & post-test design without a control group  - n=16, (93.8% male), *M*age=68.3 yrs (SD=8.4), Time since Dx= 6 yrs (median)  - led by music therapist | - Weekly 12-wk virtual group singing in small group (5-6 pairs via videoconferencing platform  - Protocol: Facial/Neck massage, oral movements, vocalizations (2 min); rhythmic warm-up/drumming (3-10 min); vocal exercises (10-15 min); therapeutic group singing (20-30 min); conversation/Check-in (5-10 min); deep breathing and/or listening to sensory based live music played by music therapist (1-2 min)  - Optional homework: listen to music provided on therapist’s website, practice specified vocal exercise | - Quality of life: PDQ-8  - Mental Health: BDI-II  - Apathy: Apathy scale;  - Satisfaction survey: 8 questions + 1 open-ended questions for feedback | - >70% adherent to the intervention  - Improvements in PDQ-8, depression & Apathy scales |
| 1. Shih   (2012)  USA  #46 | - To examine the potential therapeutic effectiveness of group singing  - Speech and language rehabilitation approach | - Pre- & post-test design without a control group  - n=13 (*M*age=66 yrs (SD=7.9); 11 males; Time since Dx=9 yrs (SD=9.5); H&Y stage=2.2  - Once weekly 90 min session for 12 wks  - led by singing instructor in collaboration with speech & language therapist | - Choral therapy sessions incl. kinaesthetic awareness, calibration to loudness and phonatory effort, increased respiratory excursion, increased vocal loudness, increased pitch range, and increased movement of articulators.  - Stretching and gross-motor movement exercises (10 min); Breathing training and structured vocal exercises (10 min); singing popular songs (70 min) | -Voice: reading rainbow passage; VHI  - QoL: V-RQoL  - Parkinson’s disability: UPDRS | -Authors suggested 12 wks of once weekly 90 min singing sessions with a trained voice/speech therapist is not helpful in improving loudness during connected speech. |
| 1. Stegemöller (2017)   USA  #47 | - To examine if singing can improve voice, respiratory pressure and QoL  - Music therapy approach | - Pre- & post-test design, comparing once a week with twice a week singing  - n=27 (*M*age=67yrs (SD=15); gender NR  - Group 1 (once a week singing): N=18;  Group 2 (twice a week singing): N=9  - Each session for 1 hr for 8 wks  - Led by music therapist | - Physical & vocal warm-ups incl. lip buzzing, glissandos, messa di voce;  - Abdominal breathing exercises; singing scales; popular song singing  - Home exercises for 30-min 2x weekly | - Respiratory function: MIP & MEP  - QoL: V-RQoL, WHOQOL  - Voice: loudness & duration of sustained vowels | - No difference between once a week and twice a week singing.  - Improvements in max. phonation time, respiratory muscle strength & QoL measures. |
| 1. Stegemöller (2020)   USA  #48 | -To assess feasibility of group therapeutic singing telehealth  -Music Therapy approach | - n=8 (Mage=74 yrs (SD=6.44), 5 males);  - Pre-recorded 8-wk singing programme using prev. protocol (2017 study)  - led by music therapist | Same as above | - Respiratory muscle strength (MIP & MEP)  - Voice: loudness & duration of sustained vowels | - Enrolment rate was 57.14%; compliance 93.75%.  - Increased respiratory muscle strengths were observed.  - Telehealth might be beneficial for people living in rural areas. |
| 1. Tamplin   (2020)  Australia  #34 | - To explore the effects of an interdisciplinary singing-based therapeutic intervention (*ParkinSong*) on voice and communication  - Music Therapy combined with speech and language therapy approach | - Participants chose to be in the singing group or control group  - Control group activities incl. painting, dancing, tai chi  - 4 groups: (a) weekly singing, (b) weekly control, (c) monthly singing & (d) monthly control groups  - Total n=75, *M*age=74.3 yrs (SD=8.2); 46 males (61%); Year since Dx=8.9 (SD=6.1); H&Y stage=2  - Weekly Singing group: N=20 (*M*age=73.3 yrs (SD=8.9); 14 males (70%)  - Weekly group sessions led by music therapist, speech therapist & allied health assistant for 12 months | - *ParkinSong* protocol  - High intensity music-based vocal exercises (30 min) incl. respiratory control, vocal loudness, pitch control activities, vocal exercises focusing on loudness, self-monitoring & speech clarification strategy practice;  - Singing popular and traditional songs (60 min)  - Social time with refreshments (30-min) | - Voice: loudness, intelligibility, Max. Phonation time  - Respiratory function: MIP; MEP  - QoL: EQ-5D, VAPP  - Assessments at baseline, 3-month & 12-month  - Not all, but some assessors were blinded to participants’ allocation | - Some improvements in vocal loudness, MEP, Voice-related QoL in weekly singing group.  - Good feasibility with low attrition rate was reported. |
| 1. Tamplin   (2023)  Australia  #49 | - To evaluate the feasibility, acceptability, and preliminary efficacy of a 12-week *ParkinSong* Online intervention on speech and wellbeing  - Music therapy combined with speech and language therapy approach | - Pre- & post-test design without a control group  - n=28 (*M*age=68 yrs (SD=7.5), Years since Dx=7.8 (SD=6.2), 16 males)  - Weekly 90-min online session for 12 weeks  - Delivered via Zoom  - Co-led by music therapist & speech and language therapist | - *ParkinSong* Online protocol based on prev. Study (2020)  - Breathing exercises and vocal singing warm-ups (15 min), speech exercises (10 min), intensive high effort singing with focus on respiratory support, vocal technique and loud vocal projection (35 min), + social time in small groups (30 min)  - Extra online resources & session recordings were available for home practice. | - Voice: loudness during conversation, maximum phonation time, syllable repetition, intelligibility  - QoL: PDQ-39, Dysarthria Impact Scale, Lille Apathy Rating Scale-Short Form  - Mental health: DASS-21  - Parkinson’s disability: MDS-UPDRS | -The recruitment rate was 90% with no attrition, adverse events, nor safety issues. The attendance was on average 89% and participants reported positive experience.  - Technology reported as the main challenge  - No improvements were seen in voice measures or wellbeing outcomes |
| 1. Tanner   (2016)  Canada  #12 | - To assess the benefits of a cross-disciplinary group singing approach to improve the vocal abilities  - Speech and language therapy + choral singing approach | - Pre- & post-test design  - n=28 (14 males),  *M*age=65.25 yrs (SD 8.3); Time since Dx= 6.3 (5.38) yrs  - Led by a speech and language therapist who is also a classical singer | - Twice a week for 12 weeks (90-min per session) + daily home practice  - Vocal exercises (35 min) incl. sustained, loud vowel sounds at various pitches and pitch glides, repetition of common phrases, melodious practice of vocal music; break (10 min); song singing (35 min) with the focus on breath support & posture | - Vocal ability: maximum phonation time, loudness, fundamental frequency range; Fundamental frequency; intelligibility | - Clinically significant improvements were observed in voice loudness (intensity range) and frequency range (semi-tones) & fundamental frequency during reading task.  - Statistical significance was detected in vocal skills (average fundamental frequency in the reading task & max intensity range). |
| 1. Yinger   (2012)  USA  #50 | - To assess the Group Music Therapy Voice Protocol on the speech  - Music therapy approach | - Pre & post design without a control group  - n=10 (*M*age=71.91 yrs (SD=9.3); 7 males; Time since Dx=8.1 yrs (SD=5.3)  - 1-hour 2x weekly group singing session for 6 weeks  - led by music therapist | - Group Music Therapy Voice Protocol  - Opening conversation (5min); physical warm-ups (5 min); breathing exercises (5 min); speech exercises (5min); vocal warm-ups (5min); singing participants’ preferred songs (20min); closing conversation (5min) | - Voice: Loudness and fundamental frequency during  reading & conversation | - Increased loudness in reading and conversational speech task.  - Participants feedback highlight that the Group Music Therapy Voice Protocol was beneficial for voice, social support and mental health. |

BDI-II=Beck Depression Inventory; CES-R=Communicative Effectiveness Survey-revised; Dx=Diagnosis; GDS=Geriatric Depression Scale; gr=Group; *M*age = Mean age; NR=Not Reported; SD= Standard Deviation; PAS=Parkinson’s Anxiety Scale; PDQ-8= Parkinson’s Disease Questionnaire-8; PDQ-39=Parkinson’s Disease Questionnaire-39; UPDRS= Unified Parkinson's Disease Rating Scale; MDS-UPDRS= MDS Unified Parkinson’s Disease Rating Scale; VHI=Voice Handicap Index; QoL=Quality of Life; V-RQoL=Voice Related Quality of life; VAPP=Voice Activity and Participation Profile; yrs=years

| **Table 2a. Quality assessment for studies with control groups** |
| --- |

| **Downs & Black Quality Checklist^23^** | **Brooks 2021**  **#32** | **Brown* 2024**  **#29** | **Butala 2022***  **#30** | **Mohseni 2023***  **#31** | **Tamplin 2020**  **#33** |
| --- | --- | --- | --- | --- | --- |
| Q1. Study question | 1 | 1 | 1 | 1 | 1 |
| Q2. Outcomes | 1 | 1 | 1 | 1 | 1 |
| Q3. Participants eligibility | 1 | 1 | 1 | 1 | 1 |
| Q4. Intervention | 1 | 1 | 1 | 1 | 1 |
| Q5. Confounders | 1 | 1 | 1 | 1 | 1 |
| Q6. Findings | 1 | 1 | 1 | 1 | 1 |
| Q7. Data distribution | 0 | 1 | 0 | 1 | 1 |
| Q8. Adverse event report | n/a | n/a | n/a | n/a | n/a |
| Q9. Drop-outs | 1 | 1 | 1 | 1 | 1 |
| Q10. Probability | 1 | 0 | 1 | 1 | 1 |
| Q11. Representative sample | 0 | 0 | 0 | 0 | 0 |
| Q12. Population | 0 | 0 | 0 | 0 | 0 |
| Q13. Setting | 1 | 1 | 1 | 1 | 1 |
| Q14. Blinding participants | 0 | 0 | 0 | 0 | 0 |
| Q15. Blinding assessors | 1 | 1 | 1 | 1 | 0 |
| Q16. Outcome reporting consistency | 0 | 0 | 0 | 0 | 0 |
| Q17. Follow-up | 1 | 0 | 1 | 1 | 1 |
| Q18. Statistical analysis | 1 | 1 | 1 | 1 | 1 |
| Q19. Compliance with intervention | 1 | 1 | 1 | 0 | 1 |
| Q20. Outcome measures | 1 | 1 | 1 | 1 | 1 |
| Q21. Participants from same population | 1 | 1 | 1 | 1 | 1 |
| Q22. Study period | 1 | 1 | 1 | 1 | 1 |
| Q23. Randomisation | 0 | 1 | 1 | 1 | 0 |
| Q24. Concealment | 0 | 0 | 0 | 1 | 0 |
| Q25. Adjustment for confounders | 1 | 0 | 1 | 1 | 1 |
| Q26. Follow-up | 1 | 0 | 1 | 1 | 1 |
| Q27. Power calculation | 0 | 0 | 0 | 1 | 0 |
| **Total score & rating** | **18 (Fair)** | **18 (Fair)** | **19 (Fair)** | **21 (Good)** | **18 (Fair)** |

1=Yes, 0=No, or Unable to determine; * RCTs; Rating: Excellent (25-27); Good (20-24); Fair (15-19)

| **Table 2b. Quality assessment for pre- & post-test studies without control** |
| --- |

| NHLBI** quality assessment | **Azekawa 2018** | **Chan 2019** | **Di Benedetto 2009** | **Elefant 2012** | **Evans 2012** | **Good 2022** | **Han 2018** | **Heneish 2001** | **Higgins 2019** | **Irons 2021** | **Lewellen**  **2020** | **Shah-Zamora**  **2024** | **Shih 2012** | **Stegemöller 2017** | **Stegemöller 2020** | **Tamplin 2023** | **Tanner 2016** | **Yinger 2012** |
| --- | --- | --- | --- | --- | --- | --- | --- | --- | --- | --- | --- | --- | --- | --- | --- | --- | --- | --- |
| Reference # | **#35** | **#36** | **#37** | **#38** | **#39** | **#40** | **#41** | **#42** | **#43** | **#8** | **#44** | **#45** | **#46** | **#47** | **#48** | **#49** | **#12** | **#50** |
| Q1. Study question | 1 | 1 | 1 | 1 | 1 | 1 | 1 | 1 | 1 | 1 | 1 | 1 | 1 | 1 | 1 | 1 | 1 | 1 |
| Q2. Eligibility | 1 | 1 | 1 | 1 | 1 | 1 | 1 | 1 | 1 | 1 | 1 | 1 | 1 | 1 | 1 | 1 | 1 | 1 |
| Q3. Representative participants | 0 | 0 | 0 | 0 | 0 | 0 | 0 | 0 | 0 | 0 | 0 | 0 | 0 | 0 | 0 | 0 | 0 | 0 |
| Q4. Criteria | 1 | 1 | 1 | 0 | 0 | 0 | 0 | 0 | 0 | 1 | 0 | 1 | 0 | 0 | 0 | 1 | 1 | 1 |
| Q5. Sample size | 0 | 0 | 0 | 0 | 0 | 0 | 0 | 0 | 0 | 1 | 0 | 0 | 0 | 0 | 0 | 0 | 0 | 0 |
| Q6. Intervention | 1 | 1 | 1 | 1 | 1 | 0 | 1 | 1 | 1 | 1 | 1 | 1 | 1 | 1 | 1 | 1 | 1 | 1 |
| Q7. Outcome measures | 1 | 1 | 1 | 1 | 1 | 1 | 1 | 1 | 1 | 1 | 1 | 1 | 1 | 1 | 1 | 1 | 1 | 1 |
| Q8. Blinding assessor | 0 | 0 | 1 | 1 | 0 | 0 | 0 | 0 | 0 | 0 | 0 | 0 | 1 | 0 | 0 | 1 | 0 | 0 |
| Q9. Loss to follow-up | 1 | 1 | 1 | 0 | 1 | 1 | 1 | 1 | 1 | 1 | 1 | 1 | 1 | 1 | 1 | 1 | 1 | 1 |
| Q10. Statistical analysis | 1 | 1 | 1 | 1 | 1 | 1 | 1 | 1 | 1 | 1 | 1 | 1 | 1 | 1 | 1 | 1 | 1 | 1 |
| Q11. interrupted time-series design | 0 | 0 | 0 | 0 | 0 | 0 | 0 | 0 | 0 | 0 | 0 | 0 | 0 | 0 | 0 | 0 | 0 | 0 |
| Q12. Individual/group-level data | n/a | n/a | n/a | n/a | n/a | n/a | n/a | n/a | n/a | n/a | n/a | n/a | n/a | n/a | n/a | n/a | n/a | n/a |
| **Total score** | **7** | **7** | **8** | **6** | **6** | **5** | **6** | **6** | **6** | **8** | **6** | **7** | **7** | **6** | **6** | **8** | **7** | **7** |

NHLBI** = National Heart, Lung, Blood Institute Study Quality Assessment Tool^24^ 1=Yes, 0=No or Unable to determine; N/A=not applicable

| **Table 3. Summary of Findings of controlled studies** |
| --- |

| **Meta-Analyses**  (k=number of included studies; n=number of participants) | **Results**  **Standardised Mean Difference (SMD), 95% Confidence Interval (CI); Heterogeneity (*I^2^*)** | **Certainty of the evidence**  **(GRADE) & comment**  **High ⊕⊕⊕⊕**  **Moderate ⊕⊕⊕◯**  **Low ⊕⊕◯◯**  **Very low ⊕◯◯◯** |
| --- | --- | --- |
| ***Primary Outcomes*** | | |
| 1. **Voice-Related Quality of Life**   (k= 3, n=79)  Butala (2022)  Mohseni (2023)  Tamplin (2020) | SMD= 0.59, 95% CI (–1.35 - 2.53), *I^2^*= 71.58%, No Publication bias  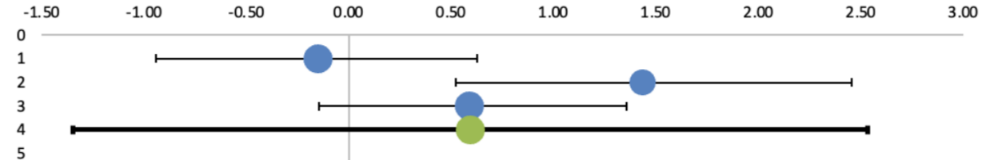 | **Very low ⊕◯◯◯**  (due to high risk of bias, inconsistency, imprecision, and indirectness) |
| ***Secondary outcomes*** | | |
| 1. **Loudness of sustained vowel**   (k=3, n=72)  Brooks (2021)  Mohseni (2023)  Tamplin (2020) | SMD = 0.67, 95% CI (0.29 - 1.05), *I^2^*= 0%, No Publication bias  Singing in favour  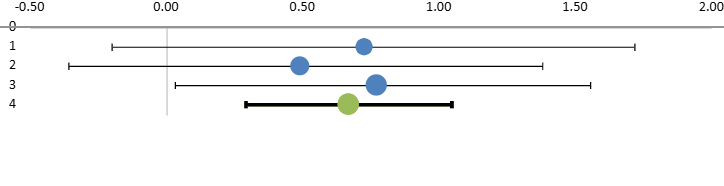 | **Very low ⊕◯◯◯**  (due to high risk of bias, imprecision and indirectness) |
| 1. **Loudness of monologue**   (k= 3, n=79)  Butala (2022)  Mohseni (2023)  Tamplin (2020) | SMD= 0.48, 95% CI (–0.17 - 1.13), *I^2^*= 0%, No Publication bias  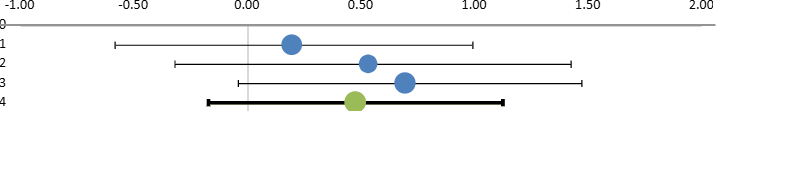 | **Very low ⊕◯◯◯**  (due to high risk of bias, imprecision and indirectness) |

| **Table 4. Summary of Findings including data from non-controlled studies** |
| --- |

| **Meta-Analyses**  (k=number of included studies; n=number of participants) | **Results**  **Standardised Mean Difference (SMD); 95% Confidence Interval (CI); Heterogeneity (I^2^)** |
| --- | --- |
| ***Primary Outcomes*** | |
| **1. Quality of life** | |
| **1.1 Voice-Related QoL**  **(V-RQoL)**  (k=10 n=127) | SMD= 0.16, 95% CI (–0.02 - 0.34), *I^2^* =0%.  Publication bias: Y (adjusted SMD= 0.07, 95% CI (–0.13 - 0.28), *I^2^* =10.79%) |
| ***Secondary outcomes*** | |
| **2. Voice** | |
| **2.1 Max. Phonation Time**  (k=11 n=157) | SMD=0.38, 95% CI (0.18 - 0.59), *I^2^*=23.48%, Publication bias: No |
| **2.2 Loudness of sustained vowel** (k=8, n=99) | SMD= 0.50, 95% CI (0.14 - 0.86), *I^2^* =50.96%, Publication bias: No |
| **2.3 Loudness of reading**  (k=10, n=143) | SMD= 0.40, 95% CI (–0.02 - 0.81), *I^2^* =69.92%, Publication bias: No |
| **2.4 Loudness of monologue**  (k=7, n=115) | SMD=0.22, 95% CI (–0.29 - 0.74), *I^2^* =78.44%, Publication bias: No |
| **2.5 Pitch range**  (k=5, n=91) | SMD=0.57, 95% CI (–0.01 - 1.15), *I^2^* =61.99%,  Publication bias: Yes (adjusted SMD= 0.48, 95% CI –0.11 - 1.08) |
| **3. Respiratory function** | |
| **3.1 Max. Inspiratory Pressure** **(MIP)** (k=4, n=65) | SMD=0.46, 95% CI (0.07 - 0.85), *I^2^*=0%, Publication bias: No |
| **3.2 Max. Expiratory Pressure (MEP)** (k=4, n=65) | SMD=0.58, 95% CI (–0.25 - 1.41), *I^2^*=68.09%, Publication bias: No |
| **4. Mental health** | |
| **4.1 Depression**  (k=7, n=163) | SMD= –0.16, 95% CI (–0.42 - 0.01), *I^2^* = 35.71%, Publication bias: No |
| **4.2 Anxiety**  (k=4, n=128) | SMD= –0.05, 95% CI (–0.57 - 0.48), *I^2^* =69.02%, Publication bias: No |
| **4.3 Stress**  (k=3, n=121) | SMD= –0.13, 95% CI (–0.75 - 0.50), *I^2^* =73.92%, Publication bias: No |

| **Singing Intervention Synthesis** |
| --- |

**Figure 2. Singing intervention contents**


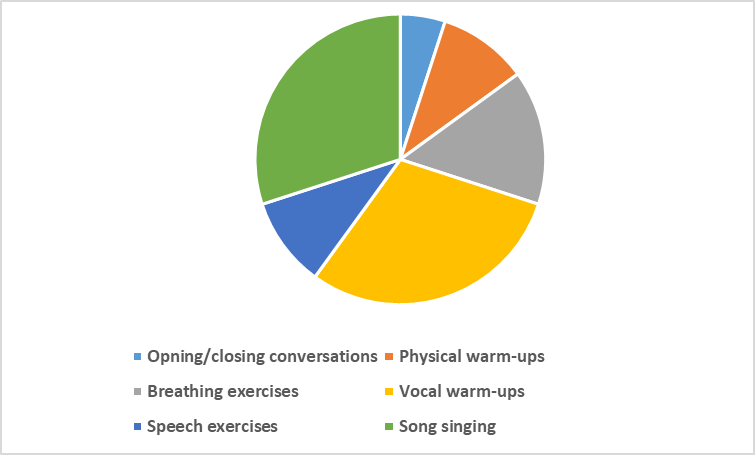


**Figure 3. Number of facilitators of the singing interventions** **by professional background**


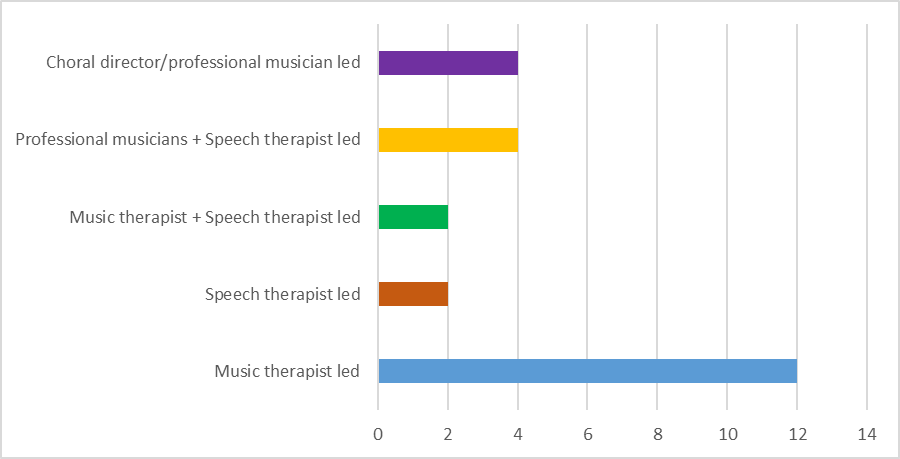

Supplement: online supplemental file 1 [file bmjopen-15-11-s001.docx]
