# Supplementary material for: Singing interventions for people living with Parkinson’s: a systematic review and meta-analysis
Source: BMJ Open. 2025 Nov 24;15(11):e089154. doi: 10.1136/bmjopen-2024-089154 (PMC12645629; doi:10.1136/bmjopen-2024-089154)
Supplement: Supplementary file 2 [file bmjopen-2024-089154.reviewer_comments.pdf]

PEER REVIEW HISTORY

BMJ Open publishes all reviews undertaken for accepted manuscripts. Reviewers are asked to complete a checklist review form and are provided with free text boxes to elaborate on their assessment. These free text comments are reproduced below.

ARTICLE DETAILS

Title (Provisional)

Singing interventions for people with living with Parkinson's: A systematic review and meta-analysis

Authors

Irons, J. Yoon; Sheffield, David

VERSION 1 - REVIEW

**Reviewer**	**1**
**Name**	**Barnish, Maxwell**
**Affiliation**	**University of Exeter, Exeter Medical School**
**Date**	**30-Jul-2024**
**COI**	**None**

This is a well-conducted systematic review and meta-analysis. I have two methodological queries:

1. Could the authors please justify the choice of Downs and Black (1998) for risk of bias assessment? It is widely considered an old tool and superseded by ROB-2 and ROBINS.

2. Could the authors please clarify how in the meta-analysis they overcame the frequently encountered issue in arts research of no measure of variation being reported for change scores (post minus pre test) when assessing mean differences?

Also, the work is not novel and is quite predictable in many ways. This is not an issue in itself for BMJ Open, however the authors would be encouraged to state more clearly how this work advances the state of scientific knowledge. The most recent review in the field (Barnish et al, 2020) recommended comparing artistic modalities. It would be useful to see what the authors consider the value of continuing to just assess singing vs non-arts interventions.

**Reviewer**	**2**
**Name**	**Dingle, Genevieve**
**Affiliation**	**The University of Queensland, School of Psychology**
**Date**	**07-Aug-2024**

COI **I have met one of the authors (Y. I) and co-authored a paper with her and numerous others following a workshop in 2018. I don't believe this represents a significant COI for this review.**

---

https://journals.sagepub.com/doi/full/10.

**Reviewer**	**3**
**Name**	**Orso, Massimiliano**
**Affiliation**	**C.R.E.A. Sanità (Centre for Applied Economic Research in Healthcare)**
**Date**	**22-Sep-2024**
**COI**	**No competing interests to declare**

Dear Editor,

thank you for giving me the opportunity to revise this paper. This systematic review and meta-analysis aims to evaluate the effectiveness of singing interventions for well-being, mental health, and communication, motor, and respiratory functions through meta-analysis, and to examine the practices used in the singing interventions.

I think it is well written and merits the publication after having addressed the following comments:

Abstract: please add details on the risk of bias assessment.

Methods:

- Protocol: page 5, line 31: I can not access the study protocol on Open Science Framework.

- 2.1 Searches: please provide as supplementary materials the full search strategies for all databases (Item #7 of PRISMA checklist).

- After 2.4 paragraph (Interventions), you should specify which are the comparators of your interest, such as no singing interventions, other types of interventions, and so on.

- paragraph 2.6 is missing, renumber the following accordingly

- Page 6, 2.10 Measures of treatment effect paragraph: did you plan to pool together results from randomized and non-randomized studies? If so, what is the rationale for doing so? I would recommend performing separate meta-analyses from RCTs and NRSI or, at least, to perform subgroup analyses for RCTs and NRSI.

- page 7, line 7: use the past (screened, not will screen)

- disagreements in the review process were resolved contacting an independent researcher: due to there are only two Authors in this review, the third researcher is external to the review Authors?

- page 7, line 18: why you cited the reference n. 25 in support of your statement?

- quality assessment: more recent and robust tools exist for QA of randomized and non-randomized studies, such as the Cochrane RoB 2 tool (RCTs) and the ROBINS-I (NRSI). However, if for the Editor the tools used are fine, for me they are ok as well.

- Reporting biases (item #14 of PRISMA checklist): did you plan to assess the risk of reporting biases? If not, why? Please explain.

- Certainty assessment (item #15 of PRISMA checklist): did you plan to assess the certainty (or confidence) in the body of evidence for each outcome, using methods such as GRADE? If not, why? Please explain.

Results:

- page 8, lines 10-15: according to PRISMA flow diagram 72 studies were excluded after full-text assessment, while here you cited only few of them. Please provide a full list of all excluded studies and the reasons for their exclusion.

- page 11 Loudness of sustained vowel: in the figure 3, the results are slightly different: 0.54 (0 - 0.99). In addition, for the two last studies (Stegemoller 2020 and Tamplin 2020) there are some data missing in the figure (CI upper limit and weights).

- in all the meta-analyses the risk of publication bias has been evaluated. In the methods section I did not find a description of the methods used to evaluate this risk. Could you add some details in the statistical analysis paragraph? Usually, it is assessed both through a visual examination of funnel plots and using appropriate statistical tests.

**Reviewer**	**4**
**Name**	**Rick, Caroline**
**Affiliation**	**University of Nottingham, Nottingham Clinical Trials Unit**
**Date**	**11-Mar-2025**
**COI**	**None**

This is a fascinating topic, and the potential for a positive impact on voice and quality of life of singing interventions for people with Parkinson's deserves careful investigation. The authors have systematically analysed all the available data and have clearly identify the limitations of the current literature and that further research in this topic is required.

Combining the existing literature on this complex behavioural intervention is extremely difficult given the differences in design and delivery of the studies, which makes further conclusions challenging.

Major:

I felt that the limited data identified in this review and the different methodologies (randomised controlled trials, before and after etc), do not require the number and complexity of the analyses undertaken and that there are risks of spurious results associated with multiple analyses of small data sets.

I was surprised that the search terms listed did not include "Parkinson" but only Parkinsonian Disorders or PD or Parkinsonism (section 2.1)

I don't follow the Authors' logic in the discussion, of the importance of different aspects of the singing sessions, given the quality, size and results presented from their systematic review. For example the authors postulate that: "From neurological aspects of breathing, deep or abdominal breathing might entrain brain electrical activity64: i.e., slow breathing engenders synchrony of

brain waves65, which results in better communication between diverse brain regions66…. a recent systematic review and meta-analysis of breathwork found that slow-paced breathing e.g., practiced in yoga, meditation and singing, has small to medium effects on reducing stress and improving mental health67." However, the results of their own systematic review were "Four studies16,44,47,59 involving 141 participants evaluated anxiety before and after the singing interventions. There was no significant improvement from pre- to post-intervention (SMD with 95% CI = -0.17 (-0.53–0.19), I 2 =37.78%). No publication bias was detected (Supplementary Table 3)."

I think the authors need to be cautious of the difference between loudness and communication, the two are not interchangeable.

Minor:

1. There seems to be a repetition of information in background: "Singing is also known to induce neurotransmitters including endorphins and oxytocin, which enhance bonding and increase pain thresholds9,10." And then 6 lines later: "Singing may enhance health and wellbeing through promoting neurochemicals such as dopamine, cortisol, serotonin, and oxytocin".

2. Clarke et al 2007 (reference 4) must have been superseded.

3. Inconsistent use of Parkinson's disease, Parkinson's, PD (acronym not defined) and PlwP throughout text.

4. Acronym RCT (acronym not defined) and randomised controlled trial used throughout text.

5. Grammar: page 10 (labelled p9) line 37 …Butala25 did not provide necessary data of from the baseline…

VERSION 1 - AUTHOR RESPONSE

Reviewer: 1
Dr. Maxwell Barnish, University of Exeter
Comments to the Author:
This is a well-conducted systematic review and meta-analysis. I have two methodological queries:

1. Could the authors please justify the choice of Downs and Black (1998) for risk of bias assessment? It is widely considered an old tool and superseded by ROB-2 and ROBINS.	We have considered ROBINS; however, ROBINS is not suited for non-RCTs, which our sys review included.
2. Could the authors please clarify how in the meta-analysis they overcame the frequently encountered issue in arts research of no measure of variation being reported for change scores (post minus pre test) when assessing mean differences?	All meta-analyses we carried out were using post-pre means and SD from the included studies. Majority of the included studies reported pre and post Mean and SD. Some authors reported *d* value (e.g., depression scores (e.g., Brooks provided *F* values; Good *t* values, which are standardised measures), which we were able to use in the analysis. We also contacted authors for data, i.e., Brown (2025) and Butala (2023).

Also, the work is not novel and is quite predictable in many ways. This is not an issue in itself for BMJ Open, however the authors would be encouraged to state more clearly how this work advances the state of scientific knowledge. The most recent review in the field (Barnish et al, 2020) recommended comparing artistic modalities. It would be useful to see what the authors consider the value of continuing to just assess singing vs non-arts interventions.	The strengths of our systematic review are:<br>(1) We've carried out most up-to-dated searches and were able to include 23 studies (20 non-controlled studies and 3 RCTs, where Brown et al. (2024) was not identified in any other systematic reviews.<br>(2) The findings provide (a) indication of the need for well-designed RCTs; (b) important estimates of the effects sizes for the future trials (before our systematic review, such estimates did not exist and funders would ask for this; (c) our systematic review also shed light on the singing intervention contents, with outcome measure recommendations.<br><br>Compared with other arts-based interventions, singing is a novel intervention, offering specific elements for people living with Parkinson's. Therefore, some of the outcome measures in singing studies would focus on specific clinical outcome measures, such as loudness of voice, which other arts-based group activities are not suitable.<br>Combining all, our current revised systematic review adds noble findings, to further our understanding of singing interventions for people living with Parkinson's.

Reviewer: 2
Dr. Genevieve Dingle, The University of Queensland
Comments to the Author:

Thank you for the opportunity to review this paper which describes a systematic review of studies related to the effects of singing for people living with Parkinson's. Twenty studies met selection criteria for the review, of which 12 studies were included in the meta-analyses. The meta-analyses found positive effects of singing for three voice measures (Maximal Phonation Time, Loudness of Sustained Vowel, Pitch Range), and a respiratory function measure. Importantly, there were no effects of singing for quality of life or mental health. Overall this is a substantial piece of work that will make a significant contribution to the literature on singing and health.

I have some suggestions below for how the authors might further develop and clarify some aspects of the manuscript.

**Abstract:** there are more subheadings than is typically the case in abstracts (**4 separate methods subheadings**) – is this necessary? The **title** mentions singing whereas the abstract mentions singing interventions – neither are defined. Could a brief definition be included here – e.g., do the interventions include both individual and group singing interventions?	**Abstract:**<br>**4 separate methods subheadings -** Yes, we've followed the Journal's format.<br>**Title** has been amended to include singing interventions.

The phrase **'limited studies'** (and **'limited design'** in the 'what this study adds' box) would be better worded as 'studies with risks of bias'. In my view, **the summary of singing intervention practices** would be better placed before the effectiveness results. The **conclusions** could say something about why no quality of life or mental health effects were found, and what elements could be included in the programs to enhance these effects.<br><br>**Minor point**: don't start sentences with a number, turn it into text, e.g., Twenty studies.	Brief definition: Yes, we included both individual and group singing interventions. Due to the word limit in Abstract, we were unable to include a definition of singing interventions, however, it's defined in the Methods – 2.4 Types of interventions.<br><br>'What this study adds' box: this box has been amended according to the Editor's advice. In the strengths and limitations section in Discussion, we included our concerns re your comments on **limited studies/limited design**, where the current review is limited by three RCTs.<br><br>**the summary of singing intervention practices:** Thank you. We've placed singing intervention practices summaries in front of the metal analysis results.<br>**Conclusions:** thank you. In general, we've amended our conclusions. The current systematic review's findings are limited to support singing interventions for PLwPs. Given there is no definitive evidence supporting for QoL and mental health, we were not able to include this aspect. More, we don't know yet what elements in the singing interventions could enhance QoL and mental health yet. Therefore, we omitted all speculative discussions around the potential benefits of singing for QoL and mental health.

	**Minor point:** thank you. If under 10, we understand that it's advised to spell the numbers, but if the numbers are above 10, we don't need to spell them out.
**Introduction:** A mix of terminology is used including Parkinson's disease and the abbreviated PD and PLwP. Suggest using Parkinson's throughout, which is the preferred term used by Parkinson's UK.<br>There is a bit of a jump from the paragraphs about Parkinson's to the paragraph about singing. I also don't see the relevance of some of the first sentences in this paragraph – e.g. virtual choir, pain thresholds and neurotransmitters are not well linked to the scope and objective of the study. This could integrate much better with the symptoms and experiences of people with Parkinson's from the previous sections.	**Introduction: Terminology:** Thank you. We've corrected this and used Parkinson's and PLwP throughout.<br><br>Thank you. We have made a better connection between the first (relating to Parkinson's) and second (singing intervention) paragraphs. We have also summarised relevant singing studies to make a better justification for this review.
**Methods:** The selection criteria make no mention of **quantitative, qualitative** or experimental methods, however, the studies presented appear to all be quantitative. Could this be clarified?<br>Is there a reason that the **protocol was published via OSF** rather than Prospero (where other scholars are likely to search for systematic review pre-registrations)?<br><br>What was the **rationale for the minimum intervention length of two weeks**?<br><br>For readers who are unfamiliar with **Downs and Black and the National Heart, Lung and Blood Institute quality assessment tool**, could a summary and number of **quality assessment** criteria be included here?<br><br>All of the usual meta-analytic indicators were included. I appreciated the tables, figures and PRSIMA checklist.	**Methods:**<br>Under the 2.2 types of studies, we've clarified this by adding 'quantitatively'.<br><br>**Protocol via OSF**: Initially, this review protocol was published with Cochrane Systematic Reviews (Irons et al. (2019). Singing for people with Parkinson's disease. The Cochrane Database of Systematic Reviews, 2019(2), CD013279.). For the current review, we have registered our modified protocol with OSF, to include non-randomised studies.<br><br>**Min intervention length of two weeks**: We based this rationale on a study, which demonstrated that older adult singers

	needed at least 2 weeks to adopt the diaphragmatic breathing method (Engen RL. The singer's breath: implications for treatment of persons with emphysema. Journal of Music Therapy 2005;42(1):20-48.) Also, a previous RCT showed a 2-week singing intervention was effective in maintaining/improving respiratory function in young people (Irons, et al. (2012). Singing Therapy for Young People With Cystic Fibrosis: A Randomized Controlled Pilot Study. *Music and Medicine*, *4*(3), 136-145.)<br><br>**Quality assessment:** Downs and Black was chosen to assess both RCTs and non-RCTs with control groups. For the pre- and post-test studies without a control group, we adopted the National Heart, Lung, and Blood Institute Quality assessment tool, as this allowed to examine key elements, such as sample size, blinding assessor, and consistency of outcome reporting. All detailed criteria are presented in Table 2a and 2b in the Supplementary files.<br><br>Thank you for your comments. Given the updated searches during the revision period, we have conducted new meta-analysis. Accordingly, all tables, figures and PRISMA checklist have been updated.

**Results:** Twenty studies met the selection criteria, including 3 RCTs, 2 controlled studies and 15 pre-post design studies. The singing interventions ran for durations between two weeks and two years. (One might question whether programs of such different durations are really suitable to be included in the same category)<br>**The plot for Figure 2** doesn't appear to have a combined average effect size?<br>**3.2 'All included studies' rationale'** is anthropomorphism – suggest rewording to 'The rationale of all studies included….'. Similarly, 'Music therapy's approaches' – suggest rewording to 'the approaches taken in singing interventions led by music therapists'…	**Results:**<br>Evans study was excluded in the analysis due to the 2-year intervention length, which was not similar to those of other studies. We have stated this in Section 3.4. Meta-analysis.<br><br>**Figure 2**: Given the new, updated searches and analysis during the revision period, we have now amended our results tables and figures.<br><br>**3.2 'All included studies' rationale':** Thank you. We have reworded to *'the approaches taken by music therapists'*
**Discussion:** The section on study **quality and common risks of bias could reference this paper on best practice research in group singing, health and wellbeing** where many of these have been described<br>https://journals.sagepub.com/doi/full/10.1177/2059204319861719<br>Some mention should be made here that longitudinal studies (controlled or pre-post design) may show no effects on quality of life or mental health outcomes due to the deteriorating course of Parkinson's, which a singing intervention may not be powerful enough to overcome. How many studies of the 20 reported on QoL and mental health of participants? Also, comment on the suitability of the measures used in the studies – is it possible that subtle benefits on mental health and quality of life may be better captured using qualitative than quantitative methods?	**Discussion:**<br>Thank you for the reference, which we have now included in Discussion.<br><br>Thank you. We have included discussion around QoL and some limitations of current QoL measures such as PDQ-39/PDQ-8 in Discussion. Table 1 provides characteristics of included studies, and their measures included. 4 studies assessed QoL using PDQ-39/PDQ-8; Mental health (depression, anxiety, stress) was assessed in 7 studies. Please see our analysis in 3.4 Meta-analyses section.<br>We agree that benefits on mental health and quality of life may be better captured using qualitative methods.

Reviewer: 3
Mr. Massimiliano Orso, C.R.E.A. Sanità (Centre for Applied Economic Research in Healthcare)
Comments to the Author:

Dear Editor,

thank you for giving me the opportunity to revise this paper. This systematic review and meta-analysis aims to evaluate the effectiveness of singing interventions for well-being, mental health, and communication, motor, and respiratory functions through meta-analysis, and to examine the practices used in the singing interventions.

I think it is well written and merits the publication after having addressed the following comments:

**Abstract:** please add details on the **risk of bias assessment**	Thank you. We've included details on the risk of bias assessment in the Abstract.
**Methods:**<br>- **Protocol**: page 5, line 31: I cannot access the study protocol on Open Science Framework.<br>- **2.1 Searches:** please provide as supplementary materials the **full search strategies** for all databases (Item #7 of PRISMA checklist).<br>- After 2.4 paragraph (Interventions), you should specify which are **the comparators** of your interest, such as no singing interventions, other types of interventions, and so on.<br>- **paragraph 2.6** is missing, renumber the following accordingly<br><br>- Page 6, 2.10 **Measures of treatment effect** paragraph: did you plan to pool together results from randomized and non-randomized studies? If so, what is the rationale for doing so? I would recommend performing separate meta-analyses from RCTs and NRSI or, at least, to perform subgroup analyses for RCTs and NRSI.<br><br>- **page 7, line 7:** use the past (screened, not will screen)	**Methods:**<br>- **Protocol**: Apologies. There was an error with the link. We've amended the link to the Protocol.<br>**2.1 Searches**<br>**- Full Search strategies** are provided in the Protocol, and we include a short summary in the text.<br>- **the comparators**<br>Usual care, non-singing comparators<br>**- Missing paragraph 2.6:** Thank you for noticing it. We have renumbered the Methods section.<br><br>- **Measures of treatment effect:**<br>Taken together comments from other reviewers, we decided to present the main meta-analysis outcomes: Controlled studies only – it was possible to conduct 3 of these meta-analyses. This was followed by pre- post- comparisons using both controlled studies (the singing arm of the studies) and uncontrolled, pre- post- studies on outcome measures. We had planned to examine RCT and NRSIs but it was only with the additional studies included following the recent searches that this was possible. But it was only possible for 3 outcomes and there were only 3 studies included in each case. Because the number of studies was so small it was not possible to conduct separate analyses or sub-analyses.

- **disagreements in the review process** were resolved contacting an independent researcher: due to there are only two Authors in this review, the third researcher is external to the review Authors?<br>- **page 7, line 18**: why you cited the reference **n. 25** in support of your statement?<br>- **quality assessment:** more recent and robust tools exist for QA of randomized and non-randomized studies, such as the Cochrane RoB 2 tool (RCTs) and the ROBINS-I (NRSI). However, if for the Editor the tools used are fine, for me they are ok as well.<br><br>- **Reporting biases** (item #14 of PRISMA checklist): did you plan to assess the risk of reporting biases? If not, why? Please explain.<br><br>- **Certainty assessment** (item #15 of PRISMA checklist): did you plan to assess the certainty (or confidence) in the body of evidence for each outcome, using methods such as **GRADE?** If not, why? Please explain.	**- page 7, line 7:** We have amended it and written in the past tense.<br>- **disagreements in the review process:** Yes, we consulted an independent researcher.<br>- **page 7, line 18: reference #25**: to provide the information of which authors we have contacted. We have now deleted the reference.<br>- **quality assessment:**<br>Thank you for your comments on risk of bias assessments. We considered using Cochrane RoB 2 tool (RCTs) and the ROBINS-I (NRSI), however, due to the heterogeneity of included studies, we found the Downs and Black checklist is suitable for our review, as it provides all necessary checks for both controlled and non-controlled studies. For non-controlled studies, we utilised the National Heart, Lung, Blood Institute Study Quality Assessment Tool, which seems to be suitable for the pre-/post-test singing studies included in this review.<br><br>- **Reporting biases:** (item #14 of PRISMA checklist) Funnel plot asymmetry was examined, along with examination of selective reporting. Additionally, we checked reported outcomes between trial registrations and published papers, where possible. In one study (Butala 2022), we found inconsistencies between the trial protocol and the published paper in relation to reporting outcomes (page 8). In other studies, we have not detected selectively reported outcomes.<br>- **Certainty assessment:** Thank you for your comments. During revision period, we have included GRADE assessment in the Summary of Findings table involving controlled studies.
**Results:**<br>- page 8, lines 10-15: according to PRISMA flow diagram 72 studies were excluded after full-text assessment, while here you cited only few of them. Please provide a full list of all excluded studies and the reasons for their exclusion.<br>- page 11 Loudness of sustained vowel: in the **figure 3**, the results are slightly different: 0.54 (0 - 0.99). In addition, for the two last studies (Stegemoller 2020 and Tamplin 2020) there are some data missing	**Results:**<br>- **PRISMA flow diagram:** Thank you. We've conducted a new/updated search since receiving the reviewers' comments and presented the updated search results in the revised manuscript.<br>**- Figure 3**: Apologies for the mistake. We've included amended/updated forest plots for the outcome measures.<br><br>**- Publication bias**

in the figure (CI upper limit and weights).<br>- in all the meta-analyses the **risk of publication bias** has been evaluated. In the methods section I did not find a description of the methods used to evaluate this risk. Could you add some details in the statistical analysis paragraph? Usually, it is assessed both through a visual examination of funnel plots and using appropriate statistical tests.	Thanks, as the reviewer suggest usually there is visual and statistical examination. We did perform both and now include info on this in the Methods section (in addition to the info that remains in the Results).

Reviewer: 4

Dr. Caroline Rick, University of Nottingham

**Comments to the Author:**<br>This is a fascinating topic, and the potential for a positive impact on voice and quality of life of singing interventions for people with Parkinson's deserves careful investigation. The authors have systematically analysed all the available data and have clearly identify the limitations of the current literature and that further research in this topic is required.<br>Combining the existing literature on this complex behavioural intervention is extremely difficult given the differences in design and delivery of the studies, which makes further conclusions challenging.	We appreciate your comments and suggestions for further improvements of this manuscript.
**Major:**<br>I felt that the limited data identified in this review and the different methodologies (randomised controlled trials, before and after etc), do not require the number and complexity of the analyses undertaken and that there are risks of spurious results associated with multiple analyses of small data sets.<br><br>I was surprised that the **search terms** listed did not include "Parkinson" but only Parkinsonian Disorders or PD or Parkinsonism (section 2.1)	**Major:**<br>We have reduced the number of analyses presented by excluding by presentations of sensitivity analysis where data from control trials were substituted into pre-post analysis. Thus, analysis now focuses on three outcomes with control data (Table 3) and pre-/post-test studies' data (Table 4). There is not enough data to draw a conclusion, but these meta-analyses provide estimates for designing RCTs in the future.<br><br>**2.1 Searches:** Thank you for noticing it. This was an oversight. We have amended 2.1 searches section, as we conducted new, updated searches for this revision.<br><br>**Discussion:**

I don't follow the Authors' logic in the **discussion,** of the importance of different aspects of the singing sessions, given the quality, size and results presented from their systematic review.<br>*For example, the authors postulate that: "From neurological aspects of breathing, deep or abdominal breathing might entrain brain electrical activity64: i.e., slow breathing engenders synchrony of brain waves65, which results in better communication between diverse brain regions66.... a recent systematic review and meta-analysis of breathwork found that slow-paced breathing e.g., practiced in yoga, meditation and singing, has small to medium effects on reducing stress and improving mental health67." However, the results of their own systematic review were "Four studies16,44,47,59 involving 141 participants evaluated anxiety before and after the singing interventions. There was no significant improvement from pre- to post-intervention (SMD with 95% CI = -0.17 (-0.53–0.19), I 2 =37.78%).* No publication bias was detected (Supplementary Table 3)."<br><br>I think the authors need to be cautious of the difference between **loudness and communication**, the two are not interchangeable.	Thank you. We have deleted speculations in the Discussion and included GRADE assessments.<br><br>**loudness and communication:** We agree and deleted communication. But we discuss assessing other aspects of communication in the Future Directions paragraph in Discussion.
**Minor:**<br>1. There seems to be a repetition of information in background: "Singing is also known to induce neurotransmitters including endorphins and oxytocin, which enhance bonding and increase pain thresholds9,10." And then 6 lines later: "Singing may enhance health and wellbeing through promoting neurochemicals such as dopamine, cortisol, serotonin, and oxytocin".<br>2. Clarke et al 2007 (reference 4) must have been superseded.<br>3. Inconsistent use of Parkinson's disease, Parkinson's, PD (acronym not defined) and PlwP throughout text.<br>4. Acronym RCT (acronym not defined) and	**Minor:**<br>1. Repetition: We have re-written Introduction and deleted repetitive information.<br>2. Clarke et al 2007 has been replaced by Rogers et al. (2017)<br>3. Inconsistence use of Parkinson's: Apologies for this error. We've amended this issue throughout text.<br>4. Thank you. We have defined RCTs in Section 2.2 Types of studies and used acronym RCTs throughout.<br>5. Grammar: Thank you for noticing this error. We've amended it.

randomised controlled trial used throughout text.<br>5. Grammar: page 10 (labelled p9) line 37 ...Butala25 did not provide necessary data of from the baseline...	

VERSION 2 - REVIEW

Reviewer **1**

Name **Barnish, Maxwell**

Affiliation **University of Exeter, Exeter Medical School**

Date **20-May-2025**

COI

I think the authors have done a very good job with the revision. I have a couple of thoughts and it's up to the editor to decide whether a further round of revision is required:

1. The author's response was that ROBINS is not suited for non-RCTs, but I thought it was specifically designed for non-RCTs. I don't have a particular issue with Downs and Black, it's a bit old, a bit simplistic, but it's still used. Maybe note as a limitation.

2. The citations in the reference list are in a mixture of styles, probably can be resolved during production process.

Reviewer **3**

Name **Orso, Massimiliano**

Affiliation **C.R.E.A. Sanità (Centre for Applied Economic Research in Healthcare)**

Date **09-Jun-2025**

COI

Thank you for having amended the manuscript and for addressing my previous comments.

Please find below some further points that need clarification:

- Protocol:

Thank you for providing the correct link. I also noticed that you published a Cochrane review protocol in 2019. Could you please clarify why you did not proceed with the full Cochrane review?

- Search strategy:

I have a few comments/questions regarding the search strategies published in the Supplementary Materials:

In the first search strategy, under the "Database" section, you wrote “PsycInfo (EMBASE)”. However, PsycINFO and EMBASE are two distinct databases. Additionally, in the Methods section you only mentioned PsycINFO.

Most of the databases appear to have been accessed via Ovid. Please confirm this and specify the platform used in both the Methods section and the supplementary tables.

CINAHL seems to have been accessed via EBSCOhost. Can you confirm? Again, please consistently state the platform used (e.g., Ovid, EBSCOhost) throughout the manuscript.

Web of Science: The search strategy reported does not appear to follow Web of Science syntax. Please clarify.

Search dates: All searches seem to start from 1999 (“Limiters - Publication Year: 1999–2025”), while in the previous version of the manuscript you stated that the search was conducted “from inception.” Please clarify whether the search period was changed during the update.

- PRISMA Flow Diagram:

The numbers in the updated PRISMA flow diagram differ substantially from those in the previously submitted version:

In the previous version, the number of records identified from databases was 2,442, while in the updated version it is 3,476. Did you identify 1,034 new records in the 15-month period between 21/12/2023 and 04/03/2025?

Reports assessed for eligibility were 92 in the previous PRISMA, but 42 + 11 = 53 in the updated version. Please clarify this reduction.

In the updated PRISMA, you report identifying 3,476 + 100 = 3,576 records, and that 659 duplicates were removed. This should result in 2,917 records to be screened, not 1,627 as currently indicated.

- GRADE Assessment:

Please provide more detailed explanations of when and why you downgraded for each domain (risk of bias, inconsistency, indirectness, imprecision, and publication bias). Some judgments are unclear:

For example, you stated that you downgraded for inconsistency when “inconsistency based on high I^2 value.” However, in Table 3 (“Summary of Findings of Controlled Studies”), you also downgraded outcomes such as Loudness of sustained vowel and Loudness of monologue, despite an I^2 = 0%.

Regarding imprecision, you wrote: “imprecision indicated by the overlapping confidence intervals.” According to GRADE methodology, this is not an appropriate criterion for imprecision. In fact, overlapping CIs may reduce concern about inconsistency, not raise concern about imprecision. Imprecision should be evaluated using the Optimal Information Size criterion, as detailed in the GRADE Handbook:

https://gdt.gradepro.org/app/handbook/handbook.html#h.ygojbnr1bi5y

- Outcome: Loudness of sustained vowel

Based on your GRADE judgments (downgrading for high risk of bias, inconsistency, imprecision, and indirectness), the overall certainty of evidence should be rated as very low, not low.

VERSION 2 - AUTHOR RESPONSE

**Reviewer: 1**<br><br>Dr. Maxwell Barnish, University of Exeter<br>Comments to the Author:<br>I think the authors have done a very good job with the revision. I have a couple of thoughts and it's up to the editor to decide whether a further round of revision is required:<br><br>1. The author's response was that ROBINS is not suited for non-RCTs, but I thought it was specifically designed for non-RCTs. I don't have a particular issue with Downs and Black, it's a bit old, a bit simplistic, but it's still used. Maybe note as a limitation.<br>2. The citations in the reference list are in a mixture of styles, probably can be resolved during production process.	Thank you very much for your kind comments.<br><br>1. ROBINS-I is for non-RCTs with comparisons. However, most of the studies we included don't have a comparator group. Therefore, ROBINS-I wasn't used.<br><br>2. Thank you. References are amended.
**Reviewer: 3**<br>**Mr. Massimiliano Orso, C.R.E.A. Sanità**<br>(Centre for Applied Economic Research in Healthcare)<br><br>Comments to the Author:<br>Thank you for having amended the manuscript and for addressing my previous comments.<br>Please find below some further points that need clarification:<br><br>**- Protocol:**<br>Thank you for providing the correct link. I also noticed that you published a Cochrane review protocol in 2019. Could you please clarify why you did not proceed with the full Cochrane review?<br><br>**- Search strategy:**<br>I have a few comments/questions regarding the search strategies published in the Supplementary Materials:<br><br>In the first search strategy, under the "Database" section, you wrote "PsycInfo (EMBASE)". However, PsycINFO and EMBASE are two	Thank you very much for your comments.<br><br>**- Protocol:**<br>Thank you. It was due to a decision made by the Movement Disorder

distinct databases. Additionally, in the Methods section you only mentioned PsycINFO.<br><br>Most of the databases appear to have been accessed via Ovid. Please confirm this and specify the platform used in both the Methods section and the supplementary tables.<br><br>CINAHL seems to have been accessed via EBSCOhost. Can you confirm? Again, please consistently state the platform used (e.g., Ovid, EBSCOhost) throughout the manuscript.<br><br>Web of Science: The search strategy reported does not appear to follow Web of Science syntax. Please clarify.<br><br>**- Search dates**: All searches seem to start from 1999 ("Limiters - Publication Year: 1999–2025"), while in the previous version of the manuscript you stated that the search was conducted "from inception." Please clarify whether the search period was changed during the update.<br><br>**- PRISMA Flow Diagram:**<br>The numbers in the updated PRISMA flow diagram differ substantially from those in the previously submitted version:<br><br>In the previous version, the number of records identified from databases was 2,442, while in the updated version it is 3,476. Did you identify 1,034 new records in the 15-month period between 21/12/2023 and 04/03/2025?<br><br>Reports assessed for eligibility were 92 in the previous PRISMA, but 42 + 11 = 53 in the updated version. Please clarify this reduction.<br><br>In the updated PRISMA, you report identifying 3,476 + 100 = 3,576 records, and that 659 duplicates were removed. This should result in 2,917 records to be screened, not 1,627 as currently indicated.<br><br>Reports assessed for eligibility were 92 in the previous PRISMA, but 42 + 11 = 53 in the updated version. Please clarify this reduction.<br><br>In the updated PRISMA, you report identifying 3,476 + 100 = 3,576 records, and that 659 duplicates were removed. This should result in 2,917 records to be screened, not 1,627 as currently indicated.<br><br>**- GRADE Assessment:**<br>Please provide more detailed explanations of when and why you downgraded for each domain (risk of bias, inconsistency,	Editorial Group.<br><br>**- Search strategy:**<br><br>As you mentioned, PsycINFO is available through various platforms, such as EBSCO and Ovid. EMBASE was a typo. We have corrected it to EBSCO (as we had intended).<br><br>Thank you. We can confirm Medline was searched through EBSCO<br><br>CINAHL was also used through EBSCO.<br><br>**Web of Science**: We have updated the search strategy for Web of Science (WoS).<br>The previous search strategy for WoS was for

indirectness, imprecision, and publication bias). Some judgments are unclear:<br><br>For example, you stated that you downgraded for inconsistency when “inconsistency based on high I^2 value.” However, in Table 3 (“Summary of Findings of Controlled Studies”), you also downgraded outcomes such as Loudness of sustained vowel and Loudness of monologue, despite an $I^2 = 0\%$.<br><br>Regarding imprecision, you wrote: “imprecision indicated by the overlapping confidence intervals.” According to GRADE methodology, this is not an appropriate criterion for imprecision. In fact, overlapping CIs may reduce concern about inconsistency, not raise concern about imprecision. Imprecision should be evaluated using the Optimal Information Size criterion, as detailed in the GRADE Handbook: https://gdt.gradepro.org/app/handbook/handbook.html#h.ygojbnr1bi5y<br><br>- Outcome: Loudness of sustained vowel<br>Based on your GRADE judgments (downgrading for high risk of bias, inconsistency, imprecision, and indirectness), the overall certainty of evidence should be rated as very low, not low.	the Ovid platform, which was used for the previous searches as indicated in the 2019 Cochrane protocol. Apologies for an oversight. We have now included the correct search strategy for the new search (April 2025) in the Supplementary file #1.<br><br>**- Search dates:** Thank you. Conducting updated searched in April 2025, we used limiter - publication from 1999. Previous searches did not find relevant singing studies from pre-1999. Hence, we used limiter this time in April.<br><br><br>**- PRISMA Flow Diagram**<br><br>Yes, there were new records were in the 15-

	month period. These new records were not always singing-related, relevant studies. E.g., there were records to do with birds singing.<br><br>In the new search, we used Rayyan, which helped us to eliminate irrelevant studies earlier on.<br><br>It seems that the boxes within the PRISMA flow chart have not been fully opened/altered during the submission process. Thus, some descriptions and numbers were not seen. We've corrected this problem.<br><br>**- GRADE Assessment:** Thank you for your advice.

	We have amended GRADE both in Table 3 and main text.<br><br>- Thank you for your advice on "Imprecision". Considering the Cochrane advice around Imprecision, we concluded that in the meta-analysis, only small number (n=72; n=79) of total participants were included which would have been fewer participants we'd expect to see in an adequately powered RCT. Give the overall small number of participants, it was not necessary to carry out the optimal information size according to the GRADE Handbook.<br><br><br>**-** Outcome: As above, we amended GRADE in Table 3 and main manuscript. All three

	outcome measures (Voice-related Quality of Life, Loudness of sustained vowel and Loudness of monologue) are graded very low.

VERSION 3 - REVIEW

**Reviewer**	**3**
**Name**	**Orso, Massimiliano**
**Affiliation**	**C.R.E.A. Sanità (Centre for Applied Economic Research in Healthcare)**
**Date**	**06-Aug-2025**
**COI**	

Thank you for having amended the manuscript and for addressing my previous comments.

Please find below some further points that require clarification or revision:

- **Search strategy**: Thank you for updating the Web of Science search strategy using the correct syntax. However, I attempted to replicate your search exactly as reported and retrieved **12,047 results**, compared to the **898 records** you report in the manuscript. This discrepancy is substantial: your reported number is more than **13 times smaller** than the actual number of records retrieved using the strategy in Supplementary File #1. Please refer to the attached file for the Web of Science search I conducted. Please clarify this large inconsistency. Transparent reporting of the **exact strategy used** is essential for reproducibility.

- **PRISMA Flow Diagram – Records removed for other reasons**: Thank you for updating the numbers in the flow diagram, particularly the count for "Records removed before screening." However, please clarify the rationale behind the exclusion of "**Records removed for other reasons (n=267)**". This category should be explicitly described in accordance with PRISMA 2020 guidelines.

- **PRISMA Flow Diagram – Final box formatting**
  In the last box of the PRISMA flowchart, the following standard wording should be used:
  Studies included in review (n=23)
  Reports of included studies (n=23)

As per PRISMA 2020 guidance, “**studies**” refer to the investigations or trials being reviewed, while “**reports**” refer to the publications (journal articles, preprints, etc.) that describe those studies. In your case, each study corresponds to a single report (23 = 23), but both fields should still be reported separately for clarity and consistency.

- **GRADE Assessment – Imprecision**
  Thank you for addressing my comments regarding inconsistency, which have now been adequately resolved.
  However, regarding **imprecision**, I would like to clarify a likely misinterpretation of my earlier comment. In the manuscript, you state:
  “Imprecision indicated by the overlapping confidence intervals.”
  According to the GRADE Handbook, this is not an appropriate justification for downgrading due to imprecision. In fact, overlapping confidence intervals may reduce concern about **inconsistency**, not raise concern about imprecision.
  In your reply, you mention the small sample sizes (e.g., n=72; n=79) and conclude that applying the Optimal Information Size (OIS) criterion was "not necessary." However, this is not consistent with GRADE methodology: **low sample size is precisely the reason to apply the OIS criterion**.
  I therefore recommend the following:
  - **Revise your explanation** to state that OIS was not met, which justifies downgrading
    for imprecision.
  - **Remove the sentence** “imprecision indicated by the overlapping confidence intervals” from the manuscript.
    This will ensure that your application of GRADE is methodologically sound and aligned with established guidance.

VERSION 3 - AUTHOR RESPONSE

Reviewer’s comments	Authors responses
Thank you for having amended the manuscript and for addressing my previous comments.<br>Please find below some further points that require clarification or revision:<br><br>- **Search strategy**: Thank you for updating the Web of Science search strategy using the correct syntax. However, I attempted to replicate your search exactly as reported and retrieved 12,047 results, compared to the 898 records you report in the manuscript. This discrepancy is substantial: your reported number is more than 13 times smaller than the actual number of records retrieved	Thank you for your previous comments and support. Thank you for further comments, which we’ve addressed. Please see below.<br><br>- **Search strategy**:<br>We apologies for unintentional errors that occurred when reporting Web of Science search strategy in Supplementary File #1.<br><br>It seems that we previously failed to report the restrictions on topics included when searching on Web of Science. Restrictions we had included Publication years, Document types, Clinical Neurology or Neurosciences (Web of Science Categories), Citation Topics (Neurodegenerative Diseases; Neuroscience; Parkinson's Disease) and Human. Unfortunately, these saved results were corrupted.

using the strategy in Supplementary File #1. Please refer to the attached file for the Web of Science search I conducted.<br>Please clarify this large inconsistency. Transparent reporting of the exact strategy used is essential for reproducibility.	So, we have re-ran these searches with similar restrictions and this resulted in 657 records. Below, we have presented the search steps and amended our manuscript, Supplementary File #1 (page 3) and Prisma flow diagram (Figure 1) accordingly. Importantly, no new studies were added nor were studies missed in the previous search.
- **PRISMA Flow Diagram –** Records removed for other reasons: Thank you for updating the numbers in the flow diagram, particularly the count for "Records removed before screening." However, please clarify the rationale behind the exclusion of "Records removed for other reasons (n=267)". This category should be explicitly described in accordance with c.	- **PRISMA Flow Diagram –** Records removed for other reasons:<br>We were just following the template and simply filling in what the PRISMA template 2020 asks. This category "Records removed for other reasons" included factors like the record's language, incomplete metadata, or being from an irrelevant source, which we based on the PRISMA guidance https://doi.org/10.1136/bmj.n160
- **PRISMA Flow Diagram – Final box formatting**<br>In the last box of the PRISMA flowchart, the following standard wording should be used:<br>Studies included in review (n=23)<br>Reports of included studies (n=23)<br>As per PRISMA 2020 guidance, "studies" refer to the investigations or trials being reviewed, while "reports" refer to the publications (journal articles, preprints, etc.) that describe those studies. In your case, each study corresponds to a single report (23 = 23), but both fields should still be reported separately for clarity and consistency.	- **PRISMA Flow Diagram – Final box formatting**<br>Thanks. We interpreted the contents of the last box incorrectly, and we have now amended it according to your advice:<br>Studies included in review (n=23)<br>Reports of included studies (n=23)
- **GRADE Assessment – Imprecision**<br>Thank you for addressing my comments regarding inconsistency, which have now been adequately resolved.<br>However, regarding imprecision, I would like to clarify a likely misinterpretation of my earlier	- **GRADE Assessment – Imprecision**<br><br>Thank you for your help with Imprecision criteria. As the Optimal Information Size (OIS) was not met in all three meta-analyses we have downgraded in each case. We have changed the wording as you have suggested on page 9 and 11, which we have highlighted.

comment. In the manuscript, you state:<br>“Imprecision indicated by the overlapping confidence intervals.”<br>According to the GRADE Handbook, this is not an appropriate justification for downgrading due to imprecision. In fact, overlapping confidence intervals may reduce concern about inconsistency, not raise concern about imprecision.<br>In your reply, you mention the small sample sizes (e.g., n=72; n=79) and conclude that applying the Optimal Information Size (OIS) criterion was "not necessary." However, this is not consistent with GRADE methodology: low sample size is precisely the reason to apply the OIS criterion.<br>I therefore recommend the following:<br><br>• Revise your explanation to state that OIS was not met, which justifies downgrading for imprecision.<br>• Remove the sentence “imprecision indicated by the overlapping confidence intervals” from the manuscript.<br>This will ensure that your application of GRADE is methodologically sound and aligned with established guidance<br><br><br>AND here’s the search:<br><br># Web of Science Search Strategy (v0.1)<br># Database: Web of Science Core Collection<br># Entitlements:<br>- WOS.SCI: 1980 to 2025<br>- WOS.AHCI: 1980 to 2025<br>- WOS.ESCI: 2020 to 2025<br>- WOS.ISTP: 1990 to 2025<br>- WOS.SSCI: 1980 to 2025<br>- WOS.ISSHP: 1990 to 2025<br># Searches:<br>1: TS=(parkinson*) Date Run: Wed Aug 06 2025 21:03:18 GMT+0200 (Ora legale<br>dell’Europa centrale) Results: 239423	Thank you. Here’s our search:<br><br>Search Query, Results<br>TS=(music*) , 232570<br>TS=(chorus*) , 8287<br>TS=(choral*) , 3471<br>TS=(choir*) , 4987<br>#1 OR #2 OR #3 OR #4 , 245425<br><br>TS=((voice* OR vocal*) NEAR/3 (exercise*)) , 744<br>TS=((voice* OR vocal*) NEAR/3 (train*)) , 2156<br>TS=((voice* OR vocal*) NEAR/3 (intervention*)) , 871<br>TS=((voice* OR vocal*) NEAR/3 (rehabilit*)) , 1142<br>TS=((voice* OR vocal*) NEAR/3 (program*)) , 1273<br>TS=((voice* OR vocal*) NEAR/3 (therap*)) , 2461<br>TS=((voice* OR vocal*) NEAR/3 (treatment*)) , 2599<br>#6 OR #7 OR #8 OR #9 OR #10 OR #11 OR #12 , 9387<br>TS=(sing*) , 5176344<br>#5 OR #13 OR #14 , 5413033<br>TS=(parkinson*) , 245239<br>#15 AND #16 , 16515<br>#15 AND #16 and 2025 or 2026 (Publication Years) , 581<br>#15 AND #16 and 2025 or 2026 (Publication Years) and Article (Document Types) , 468<br>#15 AND #16 and Article (Document Types) , 13328<br>#15 AND #16 and Article (Document Types) and Clinical Neurology or Neurosciences (Web of Science Categories , 7126<br>#15 AND #16 and Article (Document Types) and Clinical Neurology or Neurosciences (Web of Science Categories) and 1.52 Neurodegenerative Diseases or 1.5 Neuroscience (Citation Topics Meso) , 4614<br><br>#15 AND #16 and Article (Document Types) and Clinical Neurology or Neurosciences (Web of Science Categories) and 1.52 Neurodegenerative Diseases or 1.5 Neuroscience (Citation Topics Meso)

2: TS=(sing*) Date Run: Wed Aug 06 2025 21:03:32 GMT+0200 (Ora legale dell’Europa<br>centrale) Results: 5033875<br>3: TS=((voice* OR vocal*) NEAR/3 (exercise*)) Date Run: Wed Aug 06 2025 21:03:44<br>GMT+0200 (Ora legale dell’Europa centrale) Results: 698<br>4: TS=((voice* OR vocal*) NEAR/3 (train*)) Date Run: Wed Aug 06 2025 21:03:57<br>GMT+0200 (Ora legale dell’Europa centrale) Results: 2033<br>5: TS=((voice* OR vocal*) NEAR/3 (intervention*)) Date Run: Wed Aug 06 2025 21:04:10<br>GMT+0200 (Ora legale dell’Europa centrale) Results: 827<br>6: TS=((voice* OR vocal*) NEAR/3 (rehabilit*)) Date Run: Wed Aug 06 2025 21:04:25<br>GMT+0200 (Ora legale dell’Europa centrale) Results: 1075<br>7: TS=((voice* OR vocal*) NEAR/3 (program*)) Date Run: Wed Aug 06 2025 21:04:38<br>GMT+0200 (Ora legale dell’Europa centrale) Results: 1199<br>8: TS=((voice* OR vocal*) NEAR/3 (therap*)) Date Run: Wed Aug 06 2025 21:04:51<br>GMT+0200 (Ora legale dell’Europa centrale) Results: 2328<br>9: TS=((voice* OR vocal*) NEAR/3 (treatment*)) Date Run: Wed Aug 06 2025 21:05:11<br>GMT+0200 (Ora legale dell’Europa centrale) Results: 2507<br>10: #3 OR #4 OR #5 OR #6 OR #7 OR #8 OR #9 Date Run: Wed Aug 06 2025 21:05:50<br>GMT+0200 (Ora legale dell’Europa centrale) Results: 8908<br>11: TS=(choir*) Date Run: Wed Aug 06 2025 21:06:09 GMT+0200 (Ora legale dell’Europa<br>centrale) Results: 4493<br>12: TS=(choral*) Date Run: Wed Aug 06 2025 21:06:20 GMT+0200 (Ora legale dell’Europa	and 1.52.67 Parkinson's Disease (Citation Topics Micro) , 2758<br><br>#15 AND #16 and Article (Document Types) and Clinical Neurology or Neurosciences (Web of Science Categories) and 1.52 Neurodegenerative Diseases or 1.5 Neuroscience (Citation Topics Meso) and 1.52.67 Parkinson's Disease (Citation Topics Micro) and Human (Search within all fields) , 699<br><br>#15 AND #16 and Article (Document Types) and Clinical Neurology or Neurosciences (Web of Science Categories) and 1.52 Neurodegenerative Diseases or 1.5 Neuroscience (Citation Topics Meso) and 1.52.67 Parkinson's Disease (Citation Topics Micro) and Human (Search within all fields) and 1999 or 1997 or 1998 or 1995 or 1996 or 1994 or 1993 or 1992 or 1991 or 1976 (Exclude – Publication Years) , **657**

centrale) Results: 3069<br>13: TS=(chorus*) Date Run: Wed Aug 06 2025 21:06:38 GMT+0200 (Ora legale dell'Europa centrale) Results: 7796<br>14: TS=(music*) Date Run: Wed Aug 06 2025 21:06:52 GMT+0200 (Ora legale dell'Europa centrale) Results: 212339<br>15: #11 OR #12 OR #13 OR #14 Date Run: Wed Aug 06 2025 21:07:05 GMT+0200 (Ora legale dell'Europa centrale) Results: 224277<br>16: #15 OR #10 OR #2 Date Run: Wed Aug 06 2025 21:07:29 GMT+0200 (Ora legale dell'Europa centrale) Results: 5250167<br>17: #16 AND #1 Date Run: Wed Aug 06 2025 21:07:47 GMT+0200 (Ora legale dell'Europa centrale) Results: 16234<br>18: #16 AND #1 and Article (Document Types) and 2025 or 2024 or 2022 or 2023 or 2021 or 2020 or 2018 or 2019 or 2017 or 2016 or 2015 or 2014 or 2013 or 2012 or 2011 or 2010 or 2009 or 2008 or 2007 or 2006 or 2005 or 2004 or 2003 or 2002 or 2001 or 2000 or 1999 (Publication Years) Date Run: Wed Aug 06 2025 21:11:03 GMT+0200 (Ora legale dell'Europa centrale) Results: 12047	

VERSION 4 - REVIEW

Reviewer 3

Name Orso, Massimiliano

Affiliation C.R.E.A. Sanità (Centre for Applied Economic Research in Healthcare)

Date 12-Oct-2025

COI

All of my previous comments have been addressed appropriately, and I consider the outstanding issues to be resolved.

I have reviewed the updated Web of Science search strategy and confirm that the reported results are now consistent with the search logic and filters applied.

As a recommendation for future work, I suggest avoiding the use of the command AND Human (Search within all fields) in Web of Science. This is not a standardized or reliable way to restrict results to studies involving human participants. It may include irrelevant studies (e.g., animal or in vitro studies that mention "human" in the text) and exclude relevant studies where the term “human” does not explicitly appear in searchable fields.
